# Neuroinflammation in Alzheimer’s disease: pathogenic signaling, biomarkers, and emerging therapeutic interventions

**DOI:** 10.3389/fonc.2026.1908830

**Published:** 2026-10-23

**Authors:** Poonam, Rekha Rana, Mohita Thakur, Aanchal Sharma, Shivani Pannu, Shivani Sharma, Subh Naman, Sapna Rani, Diksha Devi, Vishal Kumar, Sunny Kumar

**Affiliations:** 1 Sachdeva College of Pharmacy, Gharuan, Punjab, India; 2 Desh Bhagat University, Mandi Gobindgarh, Punjab, India; 3 Rayat Bahra Institute of Pharmacy, Hoshiarpur, Punjab, India; 4 Gautam College of Pharmacy, Hamirpur, Himachal Pradesh, India; 5 Faculty of Pharmaceutical Sciences, The ICFAI University Baddi, Himachal Pradesh, India; 6 M.M College of Pharmacy, MM(DU), Mullana, Ambala, India; 7 L.R. Institute of Pharmacy, Jabli-kyar, Oachaghat, Solan, Himachal Pradesh, India; 8 National Institute of Pharmaceutical Education and Research (NIPER), Kolkata, India

**Keywords:** Alzheimer's disease, neuroinflammation, amyloid-*β*, tau, clinical trials, biomarkers, PROTACs, and targeted therapy.

## Abstract

Alzheimer’s disease (AD) is the leading cause of dementia worldwide, characterized by progressive cognitive decline and neurodegeneration. Although amyloid-β plaques and hyperphosphorylated tau are major pathological hallmarks, increasing evidence highlights persistent neuroinflammation as a critical contributor to disease progression. Activated microglia and astrocytes, inflammatory cytokines, inflammasome activation, and dysregulated immune responses promote neuronal damage and synaptic dysfunction. Similar inflammatory mechanisms are observed in neurological disorders such as multiple sclerosis (MS) and brain tumors, suggesting shared molecular pathways linking neuroinflammation, neurodegeneration, and tumor-associated immune responses. Proteomics, transcriptomics, and multi-omics approaches provide powerful strategies to characterize disease-associated molecular signatures, signaling pathways, biomarkers, and therapeutic targets. This review summarizes key neuroinflammatory mechanisms in AD, including NF-κB, NLRP3, PI3K/Akt, MAPK, cGAS–STING, mitochondrial, lysosomal, calcium, insulin, and blood–brain barrier pathways. Recent advances in proteomic and transcriptomic profiling and their integration with genomic, epigenomic, and metabolomic data are discussed for understanding disease heterogeneity and identifying clinically relevant biomarkers. Current and emerging therapeutic strategies, including anti-Aβ antibodies, anti-inflammatory agents, selective inhibitors, dual-target compounds, PROTACs, and protein–protein interaction modulators, are also evaluated. Finally, multi-omics-based precision medicine and artificial intelligence-assisted analysis may facilitate biomarker-guided patient stratification and development of effective disease-modifying therapies.

## Graphical Abstract

**Figure:**
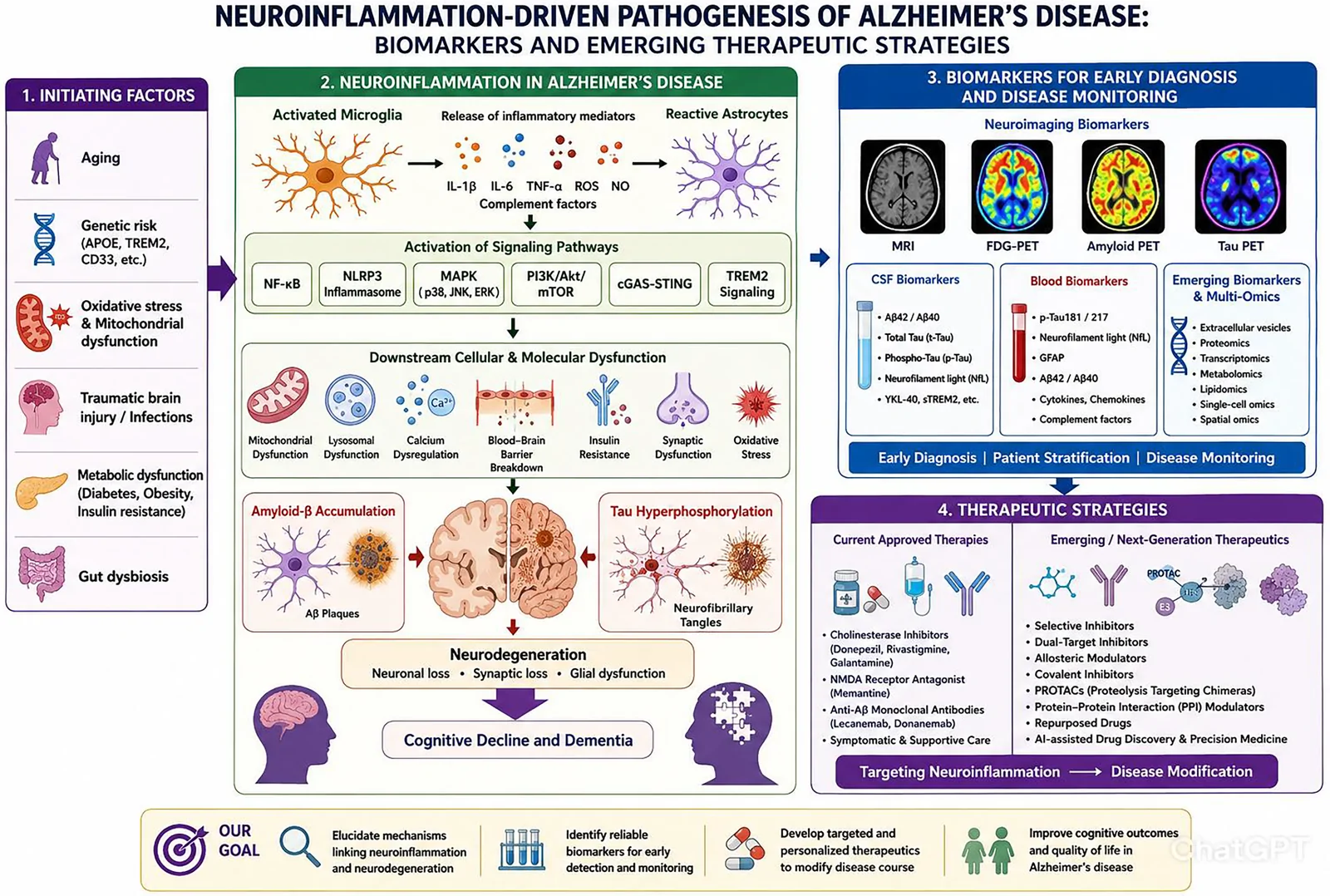
Alzheimer’s disease is driven by chronic neuroinflammation initiated by aging, genetic susceptibility, metabolic dysfunction, oxidative stress, infections, and gut dysbiosis. Activation of microglia and astrocytes triggers inflammatory signaling pathways, including NF-κB, NLRP3, PI3K/Akt, and cGAS–STING, leading to mitochondrial dysfunction, lysosomal impairment, BBB disruption, amyloid-*β* accumulation, and tau hyperphosphorylation. These pathological events promote neurodegeneration and cognitive decline. Advances in imaging, fluid biomarkers, and next-generation therapeutics offer promising opportunities for early diagnosis, disease monitoring, and precision treatment of Alzheimer’s disease.

## 1 Introduction

Alzheimer’s disease (AD) stands as the most prevalent cause of dementia worldwide, accounting for roughly 70% of all cases and presenting an escalating global health crisis as populations age. Over 55 million people worldwide already suffer from dementia, and estimates suggest that number will increase to 78 million by 2030, according to the WHO 2022 blueprint for dementia research. Dementia has a significant impact on people, caregivers, and healthcare systems around the world, as evidenced by its rising prevalence and estimated global economic burden of over US$2.8 trillion. AD is still the most prevalent and crippling type of dementia, characterized by gradual cognitive decline, functional disability, and eventually loss of independence (1, 2).

While the extracellular deposition of amyloid-*β* peptides and intraneuronal neurofibrillary tangles composed of hyperphosphorylated tau have long been recognised as the pathological hallmarks of AD, the role of immune processes has only recently moved from the sidelines to center stage. When Alois Alzheimer first described glial abnormalities alongside plaques, these observations were largely dismissed as irrelevant bystander effects (3). Today, however, a growing body of experimental, genetic, epidemiological, and neuropathological evidence positions neuroinflammation not merely as a consequence but as a driving force in disease pathogenesis. Neuroinflammatory mechanisms are also observed in other neurological disorders, including multiple sclerosis (MS) and primary brain tumors, suggesting that neuroinflammation may represent a common biological interface linking neurodegeneration, autoimmunity, and malignancy within the central nervous system (4). From the activation of microglia and astrocytes to the infiltration of peripheral immune cells and the involvement of the complement system, inflammatory mechanisms appear intertwined with every aspect of AD progression. This paradigm shift has opened new avenues for therapeutic intervention, though it also raises critical questions about timing, cell-type specificity, and the balance between protective and detrimental immune responses (5).

The inflammatory landscape of AD is remarkably complex, shaped by an intricate interplay of cellular mediators, molecular pathways, and external influences. Microglia, the brain’s resident immune sentinels, respond to Aβ deposition by releasing cytokines such as interleukin-1β, interleukin-6, and tumor necrosis factor-α, while signaling cascades involving Nuclear factor kappa-light-chain-enhancer of activated B cells (NF-Κb), p38 MAPK, Akt/mTOR, and the NLRP3 inflammasome creating a pro-inflammatory and tumor permissive environment. Among these pathways, NF-κB acts as a major transcriptional regulator of inflammatory mediators such as IL-6, TNF-α, and CCL2, while activation of p38 MAPK further promotes inflammatory cytokine production and cellular stress responses. Similarly, MS is characterized by chronic activation of microglia and astrocytes together with infiltration of peripheral immune cells, contributing to demyelination and axonal injury. In brain tumors, particularly aggressive gliomas, microglia and infiltrating macrophages are extensively reprogrammed by the tumor microenvironment and can acquire immunosuppressive and tumor-supportive functions. Thus, although the ultimate pathological outcomes differ neurodegeneration in AD, demyelination in MS and uncontrolled proliferation in brain tumors the dysregulation of CNS immune cells represents an important mechanistic connection among these disorders (4). The PI3K/AKT/mTOR pathway, which regulates cell survival, metabolism, and growth, may become dysregulated under chronic inflammatory conditions, supporting neuronal dysfunction while simultaneously promoting tumor-cell survival and proliferation. In parallel, activation of the NLRP3 inflammasome in microglia and other immune cells promotes maturation and release of interleukin-1 beta (IL-1β) and IL-18, thereby sustaining neuroinflammation and potentially enhancing immunosuppressive signaling within the TME. Importantly, these pathways are interconnected rather than acting independently; inflammatory signals can activate NF-κB and p38 MAPK, while PI3K/AKT/mTOR signaling can modulate inflammatory responses and NLRP3 activation. Thus, persistent neuroinflammation may establish a feed-forward inflammatory network that links neuronal injury with immune-cell recruitment, metabolic reprogramming, immune suppression, and tumor progression, highlighting shared signaling mechanisms between neurodegeneration and malignancy.

Recent advances in proteomics and multi-omics technologies have provided an opportunity to better understand these overlapping mechanisms at a systems level. Transcriptomic, proteomic, metabolomic, epigenomic, and single-cell approaches have revealed that neurological diseases previously considered distinct often share alterations in immune signaling, complement activation, lipid metabolism, mitochondrial dysfunction, oxidative stress, and cellular communication (6, 7). In AD, immune-related genes such as TREM2, APOE, CD33, and complement-associated factors emphasize the importance of microglial and innate immune dysfunction. Comparable immune and metabolic alterations have also been identified in MS lesions and brain tumors, supporting the existence of common molecular networks that regulate inflammation and cellular adaptation. Integrative multi-omics approaches may therefore help identify shared biomarkers and molecular signatures while also distinguishing disease-specific inflammatory states (8, 9).

Aβ plaque deposition, neurofibrillary tangles made of hyperphosphorylated tau, and extensive neurodegeneration are among the defining neuropathological characteristics of AD pathogenesis. Numerous secondary pathogenic pathways, including neuroinflammation, synaptic dysfunction, mitochondrial abnormalities, oxidative stress, and vascular impairment, are closely associated with these processes. Although it can also occur in non-amnestic forms involving linguistic, visuospatial, executive, or motor dysfunction, amnestic cognitive impairment is the most common clinical manifestation of AD (10). The clinical landscape is further complicated and illness development is accelerated by comorbid diseases like depression, diabetes, and hypertension. Effective disease-modifying treatments for AD are still elusive despite significant advancements in our understanding of its etiology. The current pharmaceutical treatments do not stop or reverse the progression of the disease; they only provide symptomatic alleviation. However, research on early identification and focused intervention has increased dramatically in recent years. The focus of therapy has changed to preclinical and moderate cognitive impairment (MCI) stages, when early intervention may have significant effects, in light of the realization that pathological alterations start decades before clinical symptoms appear (11, 12) (**Figure 1**).

**Figure 1:**
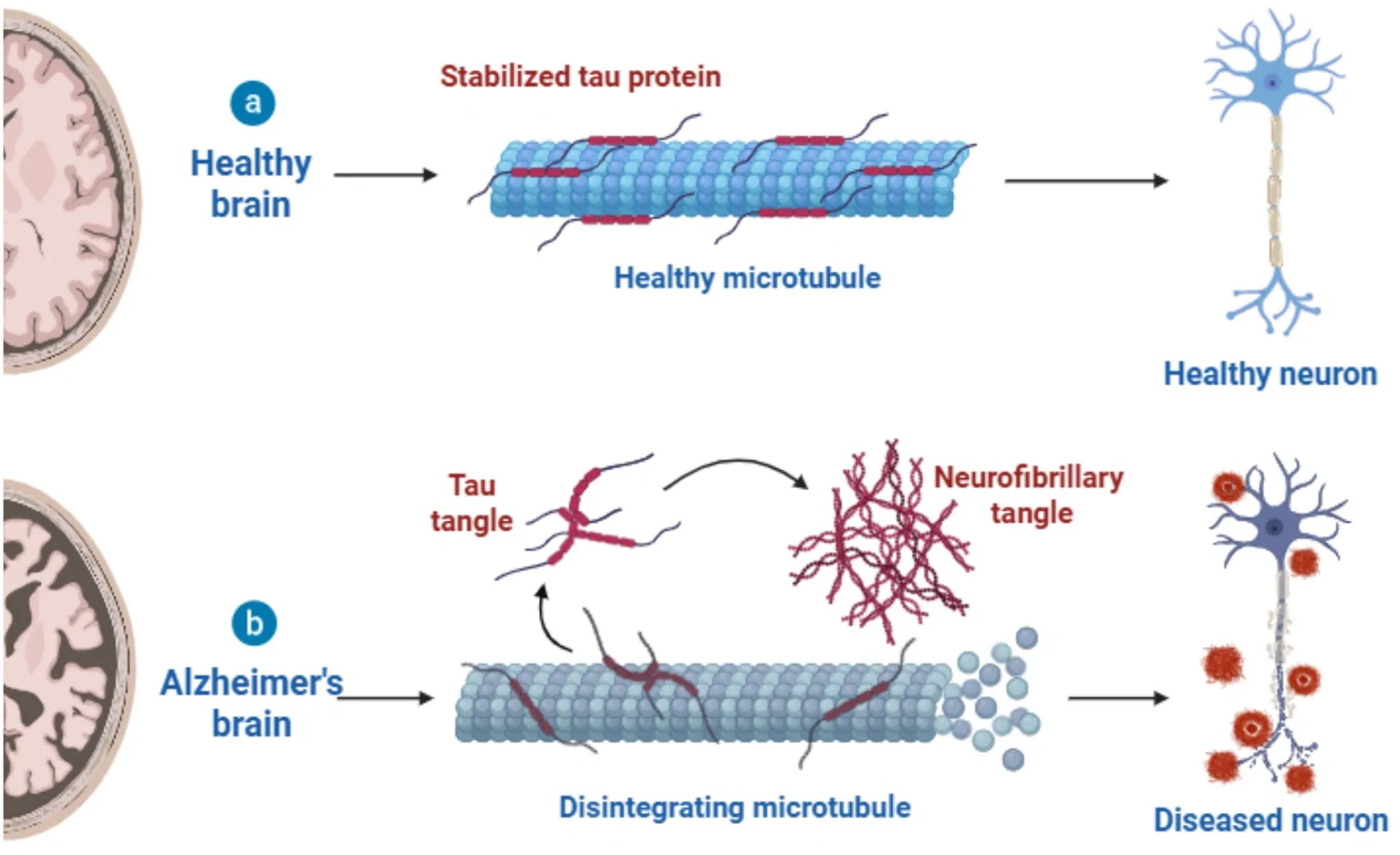
Tau functions in the normal and Alzheimer’s brain. **(A)** Tau mediates the neuron stability in the healthy brain, and **(B)** forms neurofibrillary tangles in Alzheimer’s brain.

The recognition of these shared neuroinflammatory mechanisms has important implications for biomarker discovery and therapeutic development. Proteomic and multi-omics platforms may enable the identification of common inflammatory biomarkers across AD, MS, and brain tumors, while also revealing disease-specific molecular signatures that could support early diagnosis and personalized treatment (13). In this context, biomarkers derived from cerebrospinal fluid, blood, neuroimaging, proteomics, and other multi-omics approaches are becoming increasingly important for identifying individuals at risk and monitoring disease progression. By examining AD neuroinflammation in the broader context of MS and brain tumors, this review aims to highlight convergent and disease-specific immune mechanisms and explore how shared proteomic and multi-omics signatures can advance biomarker discovery and therapeutic development. A deeper understanding of these interconnected inflammatory networks may ultimately provide new opportunities to translate knowledge across neurological diseases and develop more precise interventions for neurodegeneration, autoimmunity, and CNS malignancies (14).

## 2 Methods for this narrative review

This narrative review was conducted through a comprehensive literature search of PubMed, Web of Science, Scopus, and Google Scholar for studies published up to June, 2026. Search terms included combinations of Alzheimer’s disease, neuroinflammation, biomarkers, amyloid-*β*, tau, microglia, astrocytes, immunotherapy, drug repurposing, PROTACs, artificial intelligence, and clinical trials. Priority was given to peer-reviewed original research articles, systematic reviews, meta-analyses, clinical trials, and regulatory updates with established clinical or translational relevance. Landmark preclinical studies were included where they provided important mechanistic insights. Conference abstracts, duplicate publications, and studies lacking sufficient methodological detail were excluded. The literature was critically synthesized to provide an up-to-date overview of neuroinflammatory mechanisms, biomarker development, and emerging therapeutic strategies for AD.

## 3 Neuroinflammation and Alzheimer

Neuroinflammation is now considered one of the most important underlying mechanisms of AD, linking the early pathological events with the clinical signs of dementia. It starts with many factors and insults, such as accumulation of Aβ, infections, oxidative stress, traumatic brain injury (TBI), genetic predisposition and aging. These triggers can activate the brain’s innate immune cells, such as microglia, and activate reactive astrocytes as well. Recent studies suggest that microglia in Alzheimer’s disease (AD) cannot simply be described as “protective” or “harmful,” but instead exist in different functional states depending on disease progression. Disease-Associated Microglia (DAM) are activated around amyloid-*β* plaques and help remove and compact these plaques through enhanced phagocytosis. In contrast, Microglial Neurodegenerative Phenotype (MGnD) is associated with reduced plaque-clearing ability and increased production of inflammatory molecules that contribute to neuronal damage. The transition of microglia into the DAM state is regulated by TREM2, a receptor expressed on microglia that promotes their survival, activation, and phagocytic function. However, AD-associated TREM2 variants, such as R47H, impair this signaling, preventing microglia from efficiently clearing amyloid-*β* and leading to persistent plaque accumulation, chronic neuroinflammation, and accelerated disease progression. Upon activation, these cells release a series of inflammatory mediators including IL-1β, interleukin-6 (IL-6), tumor necrosis factor alpha (TNF-α), reactive oxygen species (ROS) and reactive nitrogen species (RNS), nitric oxide (NO), chemokines and complement proteins (15–17). These responses are protective and serve to protect, but when they are sustained, they become a chronic and destructive process. Chronic inflammatory signaling leads to blood–brain barrier (BBB) disruption and thus to infiltration of immune cells and peripheral cytokines. This disruption further exacerbates local inflammation and sets up a vicious cycle of immune activation and neuronal stress. Over time, with ongoing neuroinflammation, negative effects develop, such as chronic inflammation, early synaptic dysfunction, mitochondrial impairment and increased oxidative stress, all of which negatively impact the health and communication of the neurons (16, 18) (**Figure 2**).

**Figure 2:**
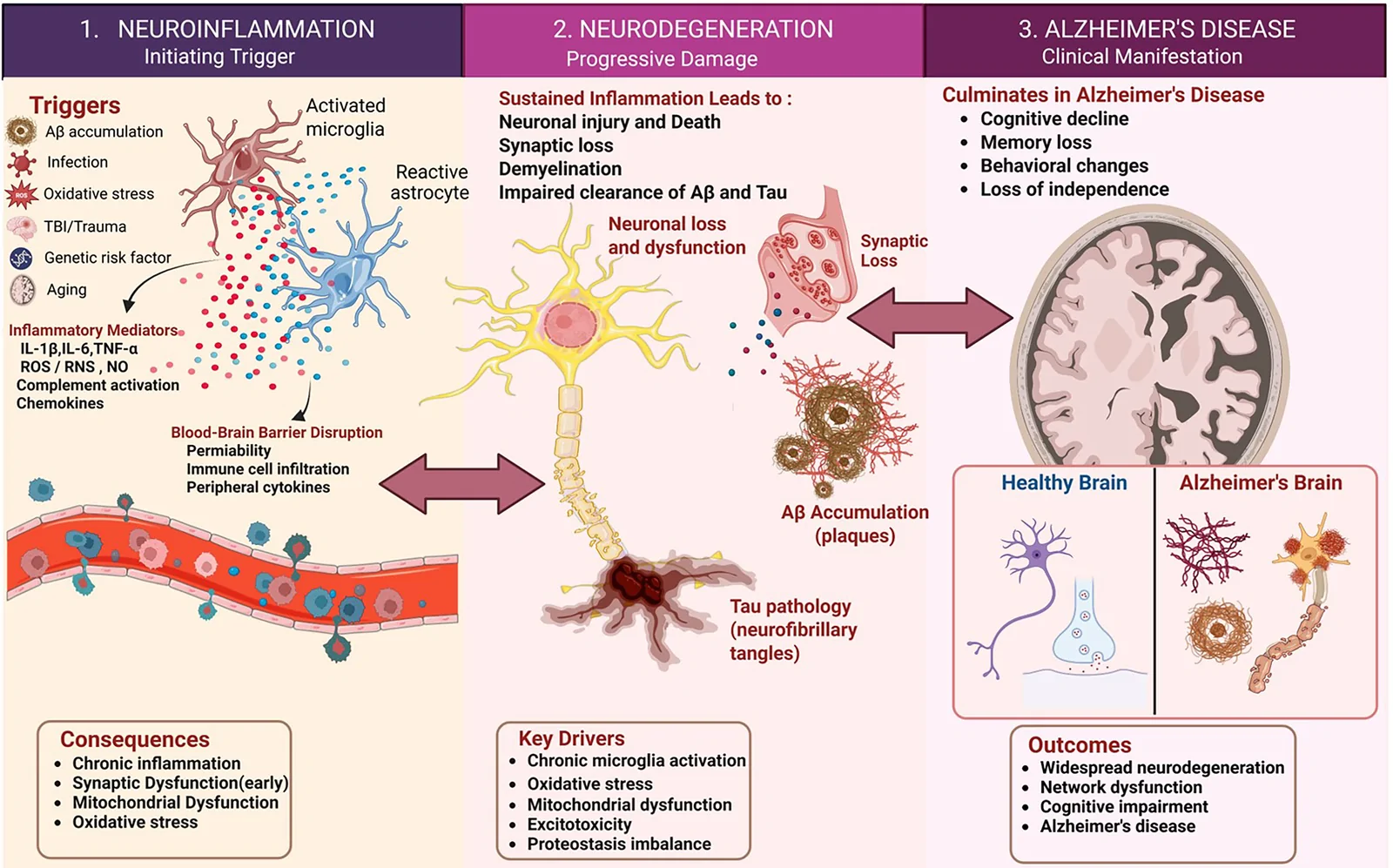
Neuroinflammation, Neurodegeneration and Alzheimer’s disease. Neuroinflammation is initiated by triggers such as amyloid-*β* accumulation, infection, oxidative stress, traumatic brain injury, genetic risk factors, and aging, leading to activation of microglia and astrocytes. Activated glial cells release pro-inflammatory cytokines, reactive oxygen species, and complement factors, causing blood–brain barrier disruption and chronic inflammatory signaling. Persistent neuroinflammation promotes neuronal injury, synaptic loss, demyelination, mitochondrial dysfunction, and impaired clearance of Aβ and tau, resulting in amyloid plaque deposition and neurofibrillary tangle formation. These pathological changes drive progressive neurodegeneration, culminating in cognitive decline, memory impairment, and the clinical manifestation of Alzheimer’s disease. The inset highlights the structural and pathological differences between healthy and Alzheimer’s brains.

However, these pathological changes lead to a neurodegenerative process that involves widespread neuronal damage and cell death, a decrease in the number of synapses, demyelination, and impaired clearance of toxic proteins like Aβ and tau. These proteins are not effectively removed and tend to accumulate in the brain, resulting in extracellular aggregates of amyloid plaques and intracellular aggregates of abnormal tau (the neurofibrillary tangles) (13, 18). These hallmark lesions disrupt neuronal communication, decrease synaptic plasticity, and play a role in the progressive dysfunction of neurons. Many of these mechanisms contribute to this degeneration, the most important being the chronic activation of microglia, which also contributes to oxidative stress, mitochondrial dysfunction, excitotoxicity and disruptions in proteostasis. These mechanisms collectively lead to widespread neuronal cell death and impairment, especially in areas of the brain involved in memory, learning and thinking. The efficient passage of nerve impulses is further compromised due to the demyelination, and the loss of synapses between neurons further weakens the communication between the neurons, causing further cognitive decline. As these pathological changes become more severe, the normal structure of the brain slowly begins to break down. Compared to a healthy brain, the Alzheimer’s brain exhibits extensive amyloid plaque deposits, tau pathology, neuronal damage, and a profound loss of tissue connections. In the end, the long-term relationship between neuroinflammation and neurodegeneration leads to AD (6, 10–12, 18, 19).

In the clinical stage, the patient experiences a progressive deterioration of cognitive function, memory loss, behavioral and personality changes and an increasing difficulty in handling everyday tasks. Patients’ attention, reasoning, problem-solving and executive function worsens and they become more reliant on caregivers. Recent proteomic studies of the human brain, cerebrospinal fluid (CSF), and plasma have greatly expanded our understanding of the molecular changes associated with AD. By integrating published human brain proteomics datasets, researchers have developed NeuroPro, a comprehensive resource for identifying proteins consistently altered across different disease stages, brain regions, tissue fractions, and pathological lesions. The updated NeuroPro v2 combines data from 53 studies and 94 analyses, covering approximately 45,440 reported protein changes involving 8,420 unique proteins. These datasets include changes associated with preclinical AD, mild cognitive impairment (MCI), advanced AD, amyloid plaques, neurofibrillary tangles, cerebral amyloid angiopathy, synaptic compartments, blood vessels, and other brain fractions. Such large-scale integration helps distinguish reproducible disease-associated proteins from study-specific changes and provides valuable candidates for understanding AD pathology, identifying biomarkers and therapeutic targets, and prioritizing proteins that may play important roles in disease progression (20–22). At the systems level, diffuse neurodegenerative changes and network dysfunction leads to widespread dysfunction in communication between regions of the brain, leading to profound cognitive impairment and functional disability. So, the pathway from neuroinflammation to neurodegeneration to AD is a continuum of pathological processes where inflammatory events that start during aging, genetic susceptibility, injury, infection, oxidative damage, and protein accumulation, lead to neuronal dysfunction, synapse loss, demyelination, plaque deposition, tangle formation, and finally to the devastating cognitive and behavioral deficits that define the disease. Knowing this interconnected pathway helps to understand the key role inflammation plays in the progression of disease and the need for therapeutic strategies to reduce neuroinflammation, maintain neuronal integrity and prevent progression from early brain injury to irreversible neurodegeneration and dementia (13, 23–25).

## 4 Shared proteomic and multi-omic mechanisms of neuroinflammation across AD, MS, and brain tumors

AD, MS, and primary brain tumors represent clinically and biologically distinct disorders, increasing evidence indicates that they converge on several interconnected molecular programs involving neuroinflammation, myeloid-cell activation, altered proteostasis, complement signaling, metabolic reprogramming, BBB dysfunction, and extracellular-matrix remodeling (26). Importantly, these shared mechanisms do not imply that the diseases have identical pathogenesis. Rather, they demonstrate that similar cellular and molecular programs can be activated in different pathological contexts and produce distinct outcomes, including synaptic dysfunction and neurodegeneration in AD, demyelination and axonal injury in MS, and immune adaptation, invasion, and tumor progression in glioma. Proteomics and multi-omics approaches are particularly valuable for identifying these convergent pathways because they allow molecular alterations to be examined across multiple biological layers, from genetic susceptibility and transcriptional regulation to protein abundance, post-translational modification, metabolism, cellular state, and tissue organization. Thus, integrating AD with MS and brain-tumor biology provides a broader framework for understanding how neuroinflammatory programs contribute to CNS disease (27).

In AD, neuroinflammation is increasingly recognized as a dynamic component of disease progression rather than merely a secondary response to A*β* and tau pathology. Brain and fluid proteomic studies have identified alterations in proteins associated with innate immunity, lipid metabolism, complement activation, oxidative stress, lysosomal function, extracellular matrix regulation, and synaptic biology (4). In particular, the interaction between apolipoprotein E (APOE) and triggering receptor expressed on myeloid cells 2 (TREM2) provides an important molecular link between lipid metabolism, microglial activation, and AD risk. TREM2 signaling promotes changes in microglial phenotype and lipid handling, whereas APOE participates in the regulation of disease-associated microglial responses around amyloid plaques. Proteomic analyses of AD brain tissue have further demonstrated that APOE genotype is associated with alterations in protein co-expression networks related to inflammation and synaptic transmission, supporting the value of systems-level proteomics for connecting genetic risk with cellular and molecular phenotypes (28).

Microglia therefore represent an important cellular intersection between AD, MS, and brain tumors (29). In AD, microglia undergo transitions from homeostatic states toward disease-associated phenotypes characterized by altered lipid metabolism, lysosomal activity, phagocytosis, inflammatory signaling, and interactions with Aβ plaques (30). Several proteins frequently detected in AD-associated microglial programs, including TREM2, TYROBP, APOE, LPL, CTSS, GPNMB, and ITGAX, reflect changes in immune surveillance and tissue-remodeling functions. These alterations are relevant to brain tumors because gliomas also profoundly remodel the resident myeloid compartment. Within glioma, tumor-associated microglia and macrophages acquire heterogeneous states that can support tumor growth, immune suppression, angiogenesis, extracellular-matrix remodeling, and invasion. Recent spatial multi-omic analysis of human gliomas demonstrated substantial re-organization of tumor–immune niches during recurrence, including a transition toward microglia- and CD206-positive macrophage-dominated environments. Such findings emphasize that spatially resolved proteomic and transcriptomic approaches can reveal disease-associated myeloid states that are not adequately represented by bulk tissue measurements (31).

The comparison between AD-associated microglia and glioma-associated myeloid cells should nevertheless be interpreted cautiously. These populations are not functionally equivalent, and their phenotypes are strongly influenced by the surrounding tissue environment. However, the recurrence of related molecular programs including lipid handling, lysosomal activity, phagocytosis, complement signaling, extracellular-matrix remodeling, and inflammatory signaling suggests that tissue-resident myeloid cells utilize a partially shared molecular repertoire in response to chronic pathological stress. In AD, these responses may initially facilitate clearance of pathological proteins but can become maladaptive and contribute to synaptic dysfunction and chronic inflammation. In glioma, analogous myeloid programs may be exploited by tumor cells to generate an immunosuppressive microenvironment and promote tumor progression. Proteomic and single-cell approaches therefore provide an opportunity to distinguish common molecular machinery from disease-specific functional consequences (32).

Complement signaling provides another important mechanistic connection among AD, MS, and brain tumors. Complement components, particularly C1q and C3, are associated with AD pathology and participate in microglia-mediated synaptic remodeling. Dysregulated complement activity can promote excessive synaptic elimination and contribute to neuronal dysfunction. In MS, complement activation occurs in inflammatory and demyelinating lesions and interacts with immune-cell recruitment, tissue injury, and myelin-associated pathology. Proteomic investigation of MS CNS tissues has identified alterations in immune, myelin, astrocytic, microglial, and inflammatory pathways, emphasizing the molecular heterogeneity of lesions. In brain tumors, complement-related signaling has also emerged as part of the interaction between malignant cells and the tumor immune microenvironment. Collectively, these observations suggest that complement is not exclusively associated with a particular CNS disease but represents a context-dependent inflammatory pathway whose biological consequences depend on the cellular and tissue environment (33, 34).

The relationship between inflammation and neurodegeneration is particularly informative when AD is compared with MS. MS is classically considered an immune-mediated demyelinating disease, but neurodegeneration and progressive axonal injury are now recognized as major determinants of long-term disability. This creates a biological continuum between inflammation and neuronal degeneration that is also highly relevant to AD. Proteomic studies of MS have identified alterations in the innate immune system, acute-phase proteins, oxidative-stress proteins, immunoglobulin chains, myelin-associated proteins, and astrocytic and microglial markers. A comparative proteomic analysis of AD and MS reported overlapping inflammatory signatures dominated by innate immune, acute-phase, and oxidative-stress proteins, while immunoglobulin chains were particularly characteristic of MS. Notably, chitinase-3-like protein 1 (CHI3L1/YKL-40) was consistently increased across multiple MS and AD studies, illustrating how proteomics can identify inflammatory molecules shared between apparently distinct neurological disorders (35–37).

Multi-omics studies further strengthen this connection by demonstrating that inflammation and neurodegeneration cannot be fully understood from a single molecular layer. In MS, integration of genomics, transcriptomics, proteomics, and metabolomics has been used to connect genetic susceptibility and transcriptional regulation with protein-level and metabolic phenotypes. Recent integrative studies have combined protein quantitative trait loci, proteome-wide association analyses, Mendelian randomization, colocalization, transcriptomics, and single-cell datasets to identify proteins potentially involved in MS progression and therapeutic response. Such approaches are particularly valuable because inflammatory activity and neurodegenerative progression may occur simultaneously but are not necessarily driven by identical molecular mechanisms (38, 39). A similar principle is increasingly applicable to AD. Genomic studies have identified risk loci associated with microglial and immune pathways, whereas transcriptomic and single-cell studies have resolved distinct cellular states in microglia, astrocytes, oligodendrocytes, endothelial cells, and neurons (40). Proteomics adds an important functional layer by measuring the proteins and protein networks that ultimately execute these cellular programs. Metabolomics and lipidomics further extend this framework by identifying alterations in mitochondrial function, lipid handling, oxidative stress, and energy metabolism. Consequently, integrated multi-omics approaches can be conceptualized as a molecular continuum: genetic variation influences cellular regulatory programs, transcriptional changes modify protein expression, post-translational regulation alters protein activity, and metabolic remodeling determines the functional state of cells within the pathological microenvironment (41).

This systems-level framework is also highly relevant to MS. Tissue and single-cell/nucleus omics studies have demonstrated substantial heterogeneity among MS lesions, with distinct microglial, astrocytic, oligodendroglial, and neuronal responses across white- and grey-matter compartments. Proteomic studies have identified changes in apolipoproteins, heat-shock proteins, myelin-associated proteins, HLA-associated molecules, and inflammatory signaling pathways. These findings highlight an important concept that also applies to AD: neuroinflammation is spatially and temporally heterogeneous. Therefore, bulk proteomic measurements from CSF, plasma, or whole-brain tissue may obscure disease-associated cell states and lesion-specific molecular programs. Combining proteomics with single-cell and spatial transcriptomics/proteomics can overcome some of these limitations by resolving which cell populations produce particular inflammatory proteins and where these proteins are localized within diseased tissue (42, 43).

Brain tumors provide an additional and particularly informative model of how the CNS inflammatory environment can be remodelled. Gliomas develop within a highly complex microenvironment containing malignant cells, resident microglia, infiltrating macrophages, astrocytes, endothelial cells, lymphocytes, and extracellular-matrix components. The tumor actively modifies these cellular populations through cytokine signaling, metabolic competition, extracellular vesicles, and cell–cell interactions. Recent spatial proteomic and transcriptomic analyses of human gliomas have demonstrated that tumor grade, recurrence, immune composition, and clinical outcome are associated with spatially organized molecular niches. The observation that recurrent gliomas can shift toward microglia- and macrophage-dominated environments illustrates how chronic tissue stress can drive stable changes in myeloid-cell states (44, 45).

This tumor-associated inflammatory remodeling provides a useful comparison with AD. In both diseases, microglia respond to persistent pathological stimuli and undergo substantial changes in lipid metabolism, lysosomal function, phagocytosis, inflammatory signaling, and interactions with neighboring cells. However, the functional consequences differ. In AD, persistent microglial activation can contribute to synaptic dysfunction, complement-mediated synaptic elimination, oxidative stress, and neuronal injury. In glioma, tumor-conditioned myeloid states can facilitate immune suppression, angiogenesis, matrix remodeling, and malignant progression. The comparison therefore does not suggest a common disease mechanism in the strict sense; rather, it identifies a conserved cellular response system that is differentially reprogrammed by disease-specific signals (29, 46, 47).

Another important shared feature is BBB dysfunction. In AD, vascular and BBB abnormalities can increase exposure of the CNS to circulating inflammatory mediators and alter clearance of pathological proteins. In MS, inflammatory infiltration across the BBB is a central component of lesion formation, whereas gliomas extensively remodel the blood–tumor barrier and vascular microenvironment to support tumor growth. Proteomic analysis of CSF and plasma can therefore provide complementary information about systemic and CNS-localized inflammatory processes. Integration of fluid proteomics with tissue, transcriptomic, and spatial data may help distinguish systemic inflammation from compartmentalized CNS responses (40, 48).

From a biomarker perspective, the AD–MS–brain tumor comparison also highlights the limitations of single-analyte measurements. Neurofilament light chain (NfL), glial fibrillary acidic protein (GFAP), CHI3L1, complement proteins, apolipoproteins, and inflammatory mediators may reflect overlapping aspects of glial activation, axonal injury, or tissue remodeling, but their interpretation depends strongly on disease context. In MS, NfL is associated with neuroaxonal injury and inflammatory activity, whereas GFAP is increasingly investigated as a marker of astroglial activation and disease progression. Similar proteins can also be altered in other neurological disorders, emphasizing the need for disease-specific molecular panels rather than reliance on individual biomarkers. Recent MS biomarker studies increasingly support multi-biomarker approaches for distinguishing acute inflammatory injury from chronic neurodegenerative processes (49, 50). Taken together, these findings establish a broader conceptual framework in which AD, MS, and brain tumors can be investigated as distinct pathological systems that converge on partially overlapping neuroinflammatory networks. Proteomics provides a direct view of altered protein abundance and cellular pathways, whereas transcriptomics, genomics, metabolomics, lipidomics, and spatial technologies provide complementary information regarding upstream regulation, cellular state, metabolism, and tissue organization. The integration of these datasets may therefore reveal how common molecular pathways including TREM2–APOE signaling, complement activation, cytokine networks, oxidative stress, lipid metabolism, proteostasis, lysosomal function, and extracellular-matrix remodeling are differentially regulated across CNS diseases (40). **Table 1** enlist the Comparative proteomic and multi-omics names across AD, multiple sclerosis (MS), and primary brain tumors on the basis of Molecular pathways.

**Table 1:** Comparative proteomic and multi-omics signatures across AD, MS, and primary brain tumors based on molecular pathways.

S. no	Alzheimer disease	Multiple sclerosis	Primary brain tumor	Interpretation
Neuroinflammation				
1.	Chronic microglial and astrocytic activation associated with disease progression and neurodegeneration	Major feature of inflammatory lesions and demyelination	Shapes tumor–immune interactions and tumor progression	Shared core process
Microglial/myeloid activation				
2.	Disease-associated microglia show changes in lipid metabolism, lysosomal function and phagocytosis	Activated microglia contribute to inflammation, demyelination and neurodegeneration	Tumor-associated microglia/macrophages contribute to immune suppression, angiogenesis and invasion	Shared, but functions differ
TREM2–APOE axis				
3.	Strongly associated with microglial activation, lipid handling and AD-related risk	Not a defining MS pathway	Related myeloid lipid-handling programs may occur in the TME	Primarily AD-associated
CHI3L1/YKL-40				
4.	Associated with neuroinflammation and glial activation	Increased in CSF and associated with inflammatory activity and disease progression	May reflect inflammatory and TME activity	Shared, context-dependent biomarker
NfL (NEFL)				
5.	Reflects neuroaxonal injury and disease-related neuronal damage	Associated with neuroaxonal injury and inflammatory disease activity	May increase with neural/tissue injury in selected tumor settings	Shared injury marker; context-dependent
GFAP				
6.	Reflects astrocyte activation and neuroinflammatory changes	Associated with astro-glial activation and disease progression	May reflect astrocytic components or astrocytic tumor biology	Shared, but biologically different
Complement signaling (C1q, C3)				
7.	Promotes microglial synaptic remodeling and excessive synaptic elimination	Associated with inflammatory and demyelinating lesion processes	Participates in tumor–immune microenvironment interactions	Shared immune pathway
Lipid metabolism				
8.	Closely linked to APOE/TREM2 signaling and disease-associated microglia	Alterations in lipid and myelin-related metabolism occur during disease	Altered lipid metabolism occurs in tumor cells and tumor-associated myeloid cells	Shared metabolic pathway
Lysosomal/proteostasis pathways				
9.	Altered protein degradation and lysosomal function contribute to microglial dysfunction and protein pathology	Altered in inflammatory and glial responses	Important for tumor-associated myeloid-cell function and tumor adaptation	Shared pathway with different consequences
Oxidative stress/mitochondrial dysfunction				
10.	Associated with neuronal injury, mitochondrial impairment and chronic inflammation	Associated with inflammatory damage and axonal injury	Can support tumor-cell adaptation and immune-cell responses	Shared stress response
Myelin-associated proteins (MBP, PLP1, MOBP)				
11.	Not a primary molecular feature of AD	Strongly associated with myelin damage and demyelinating lesions	Not a defining feature of most brain tumors	Primarily MS-associated
Tumor-associated immune programs				
12.	Not a defining feature	Not a defining feature	Includes macrophage/microglial enrichment, immune suppression, angiogenesis and invasion	Brain Tumor associated
Proteomic/multi-omics integration				
13.	Genomics, transcriptomics, proteomics, metabolomics and spatial approaches identify immune, lipid, synaptic and metabolic networks	Proteomics combined with transcriptomic/genetic data identifies disease activity and progression signatures	Spatial proteomics, transcriptomics and glycomics define tumor grade, immune niches and recurrence-associated changes	Core cross-disease framework

The above-discussed mechanisms explain the comparative proteomic and multi-omics signatures across AD, MS, and primary brain tumors.

Importantly, the major value of this cross-disease perspective is not to suggest that AD, MS, and brain tumors share a single pathological mechanism. Instead, it provides a framework for distinguishing conserved inflammatory programs from disease-specific molecular consequences. In AD, these programs are closely associated with protein aggregation, synaptic dysfunction, and progressive neurodegeneration; in MS, with autoimmune inflammation, demyelination, and chronic axonal injury; and in brain tumors, with tumor–immune interactions, immune evasion, invasion, and tumor progression. Future studies combining longitudinal proteomics with single-cell and spatial multi-omics will be essential for determining how these inflammatory states evolve over disease progression and treatment. Such integrative approaches may ultimately identify molecular signatures capable of distinguishing protective from pathogenic inflammation and enable precision strategies that target disease-associated cellular states rather than inflammation as a nonspecific process (40, 51–53).

## 5 Signaling pathways associated to AD pathogenesis

### 5.1 Neuroinflammatory signaling

Chronic inflammation and immune imbalance are common links between neurodegenerative diseases and cancer. In neurodegeneration, continuous activation of microglia and astrocytes increases the release of inflammatory mediators such as IL-1β, TNF-α, IL-6, ROS, and NO, which can promote neuronal damage. Similar inflammatory signaling occurs in the tumor microenvironment (TME), where pathways such as NF-κB and NLRP3 inflammasome promote tumor growth, immune suppression, and treatment resistance. Changes in immune-cell behavior also contribute to both diseases, including M1/M2 macrophage polarization and T-cell exhaustion, which can reduce effective immune responses. In addition, neuronal signals such as CGRP, substance P, acetylcholine, dopamine, NGF, and BDNF can influence immune-cell activity and tumor progression. Recent evidence suggests that cancer-associated peripheral nerve degeneration (CAPND) can further alter the TME by promoting IL-6-driven inflammation, M2 macrophage and Treg accumulation, reduced CD8+ T-cell infiltration, and poor response to anti-PD-1 therapy. Other common regulators, including Hsp90, TP53, PTEN, PIN1, and BCL2, show opposite or context-dependent effects in neurodegeneration and cancer (54–57).

In AD, pathological factors such as Aβ accumulation, oxidative stress, and elevated pro-inflammatory cytokines trigger chronic activation of microglia through signaling pathways including MAPK, NF-κB, and PI3K/Akt (58). This activation enhances the release of inflammatory mediators like TNF-α, IL-1β, and nitric oxide, leading to progressive neuronal damage and cognitive decline. Dysregulated microRNAs, particularly miR-25802, may further intensify neuroinflammation by promoting pro-inflammatory microglial polarization via the KLF4/NF-κB axis. Persistent NF-κB activation also stimulates the NLRP3 inflammasome, resulting in caspase-1 activation, gasdermin D cleavage, pyroptotic cell death, and release of IL-1β and IL-18, thereby amplifying inflammatory injury. Increasing evidence also links these inflammatory pathways to tau pathology, where cytokine-driven NF-κB signaling enhances tau transcription, aggregation, and phosphorylation, while tau itself can further activate microglial inflammation through TLR2/MyD88/NF-κB signaling (59, 60). Similar inflammatory mechanisms are observed in MS, where activated microglia, cytokines, oxidative stress, and peripheral immune-cell infiltration predominantly drive oligodendrocyte damage, demyelination and axonal degeneration, whereas in brain tumors, these pathways can establish an immunosuppressive tumor microenvironment that induce angiogenesis, immune evasion and tumor progression. Therefore, integrating proteomics and multi-omics approaches can help identify shared neuroinflammatory pathways and biomarkers across these CNS disorders while revealing disease-specific molecular signatures, ultimately supporting the development of targeted and precision therapeutic strategies.

Additionally, abnormal cytoplasmic DNA accumulation activates the cGAS–STING pathway in microglia, promoting interferon-mediated inflammatory responses. Neuroinflammation is further intensified through interactions between microglia, astrocytes, neurons, and infiltrating peripheral immune cells such as CD8+ T cells, which may contribute to synaptic dysfunction and neurodegeneration through the CXCL10/CXCR3 axis (61, 62). Conversely, TREM2 appears to exert protective effects by modulating the PI3K/Akt/FoxO3a pathway, suppressing inflammatory cytokine production, and limiting complement-mediated synaptic loss, highlighting its therapeutic potential in AD (63) (**Figure 3**).

**Figure 3:**
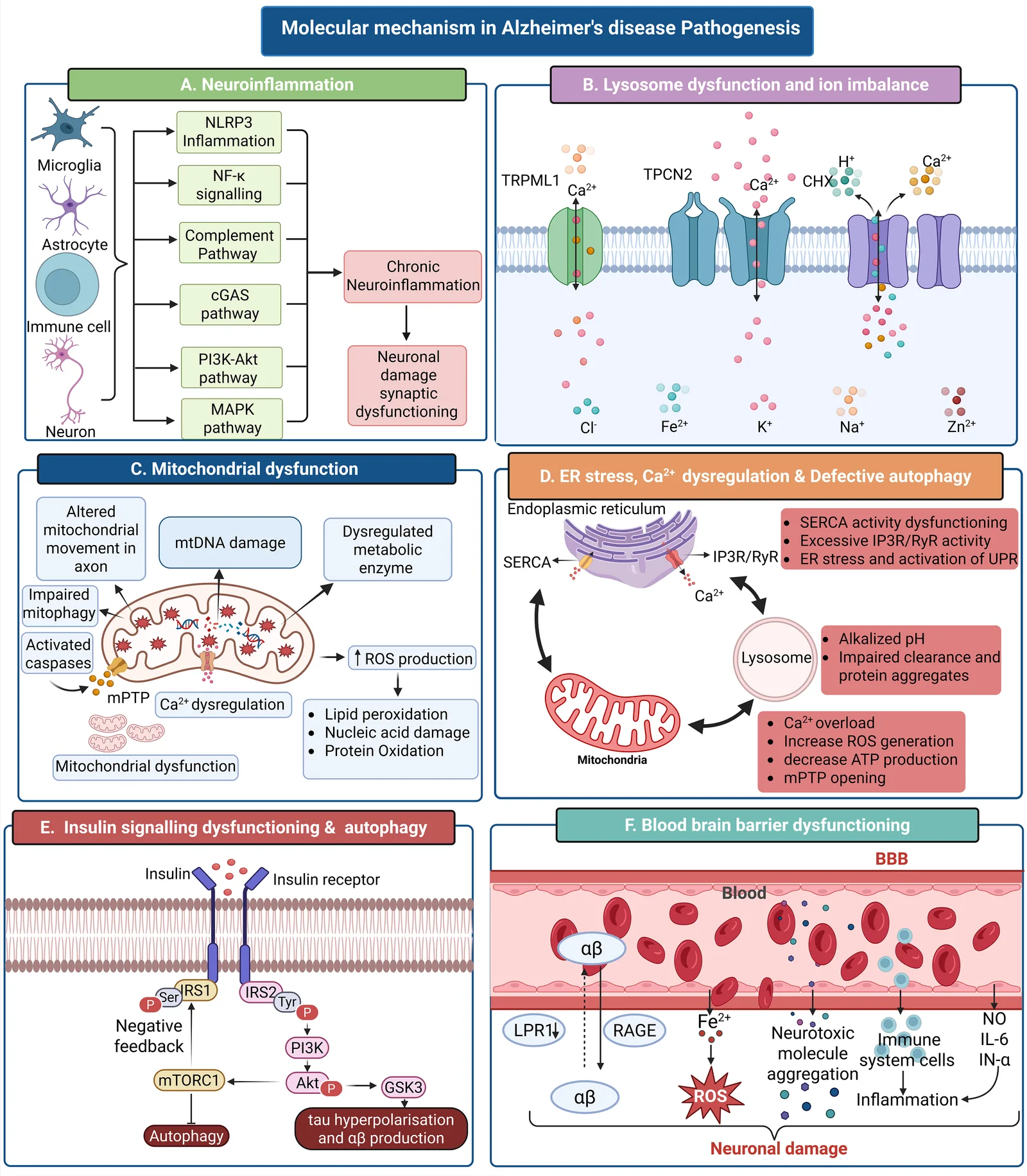
Signaling pathways involved in Alzheimer’s disease (AD) pathogenesis. **(A)** Neuroinflammatory signaling: Crosstalk among microglia, astrocytes, neurons, and other brain cells activates inflammatory pathways, leading to the release of pro-inflammatory mediators, neuronal injury, and progression of AD pathology. **(B)** Lysosomal dysfunction: Impaired lysosomal acidification and disrupted calcium homeostasis contribute to defective degradation pathways, although the underlying mechanisms remain incompletely understood. **(C)** Mitochondrial dysfunction: Alterations in oxidative phosphorylation, calcium handling, mitochondrial DNA integrity, dynamics, axonal transport, and mitophagy result in energy deficits and oxidative stress. **(D)** Calcium signaling dysregulation: Elevated cytosolic Ca^2+^ levels disrupt endoplasmic reticulum function, mitochondrial metabolism, and lysosomal homeostasis, promoting neuronal dysfunction. **(E)** Insulin signaling impairment: Defective insulin signaling contributes to metabolic dysfunction, synaptic deficits, and neurodegeneration. **(F)** Blood–brain barrier (BBB) dysfunction: Loss of BBB integrity and altered transport functions facilitate abnormal molecular trafficking, exacerbating neuronal damage and AD progression.

### 5.2 Lysosomal dysfunction

Lysosomes are essential cellular organelles responsible for degrading and recycling intracellular waste through a wide range of acidic hydrolases, thereby maintaining cellular homeostasis. In AD, lysosomal dysfunction has emerged as a major contributor to disease progression, characterized by impaired acidification, defective enzyme activity, membrane instability, disrupted ion balance, and impaired fusion of autophagosomes or endosomes with lysosomes. These abnormalities compromise the autophagy–lysosomal and endosomal–lysosomal pathways, leading to the accumulation of toxic proteins such as Aβ and relevant to multiple sclerosis (MS), where impaired cellular clearance and immune-cell function may contribute to inflammation, demyelination, and neurodegeneration, while in brain tumors, altered lysosomal function can support metabolic adaptation, tumor survival, invasion, and therapy resistance. Thus, lysosomal dysfunction may represent a shared cellular mechanism connecting impaired protein clearance, inflammation, and disease progression across CNS disorders (64, 65). Proper lysosomal acidity is mainly maintained by the V-ATPase proton pump, while ion channels regulating Ca2+, Cl−, and Na+ also contribute to lysosomal pH balance. Defective lysosomal acidification in AD reduces Aβ clearance and promotes plaque formation, suggesting that lysosomal impairment may occur early in disease development. Mutations or deficiencies in presenilin-1 (PS1) have been linked to disrupted V-ATPase maturation, altered calcium homeostasis, and lysosomal alkalinization through channels such as TRPML1 and TPCN2 (66, 67). Additionally, abnormal phosphorylation of APP β-CTF has been shown to inhibit V-ATPase assembly and activity, further increasing lysosomal pH and weakening degradative function. Together, these findings highlight the complex relationship between lysosomal dysfunction, calcium dysregulation, and impaired protein clearance in AD pathology (68).

### 5.3 Mitochondrial dysfunction

Mitochondria are essential for cellular energy production and regulate several critical processes, including redox balance, calcium signaling, and apoptosis. In AD, mitochondrial dysfunction is a major pathological feature and is associated with reduced glucose metabolism, impaired energy production, oxidative stress, calcium imbalance, and altered mitochondrial dynamics. Amyloid-*β* directly disrupts mitochondrial function by inhibiting key metabolic enzymes such as complex IV, pyruvate dehydrogenase, and α-ketoglutarate dehydrogenase, resulting in decreased ATP generation and excessive reactive oxygen species (ROS) production. Aβ also interacts with mitochondrial ABAD, further enhancing oxidative damage (69, 70). Elevated ROS levels damage mitochondrial DNA, lipids, and proteins, worsening neuronal injury and apoptosis. Disturbances in calcium homeostasis further contribute to mitochondrial damage, as Aβ increases intracellular calcium influx and promotes mitochondrial calcium overload, leading to activation of the mitochondrial permeability transition pore and subsequent oxidative stress and cell death. Hyperphosphorylated tau additionally impairs mitochondrial transport along axons, affecting neuronal energy supply and synaptic function (25, 71). Defects in mitochondrial fission, fusion, and mitophagy are also commonly observed in AD, with altered expression of proteins such as Drp1, Opa1, MFN1/2, and Fis1 promoting excessive mitochondrial fragmentation. Impaired PINK1/parkin-mediated mitophagy prevents efficient clearance of damaged mitochondria, allowing dysfunctional organelles to accumulate and further accelerate Aβ deposition, tau pathology, synaptic dysfunction, and neurodegeneration (72).

### 5.4 Calcium signaling

Calcium signaling is essential for normal neuronal functions such as gene regulation, neurotransmitter release, membrane excitability, and synaptic plasticity, with intracellular calcium levels tightly controlled by channels, transporters, and calcium-binding proteins. In AD, disruption of calcium homeostasis is a key pathological event that contributes to neuronal dysfunction and degeneration (61, 73). Aβ and tau proteins enhance the activity of L-type voltage-gated calcium channels and ligand-gated receptors such as NMDARs and α7-nAChRs, leading to excessive calcium influx into neurons. This abnormal calcium overload activates downstream neurotoxic pathways, resulting in dendritic spine loss, impaired synaptic plasticity, disrupted neurotransmission, and cognitive decline. Dysregulated calcium signaling also affects intracellular organelles including the endoplasmic reticulum (ER), mitochondria, and lysosomes (71, 74). Impaired SERCA activity and overactivation of ER calcium-release channels like IP3Rs and ryanodine receptors trigger ER stress and prolonged unfolded protein response signaling, which may ultimately induce apoptosis (75). Increased calcium transfer between the ER and mitochondria through mitochondria-associated membranes further promotes mitochondrial calcium overload, reduced ATP production, oxidative stress, and apoptotic signaling. In addition, excessive ER-derived calcium release can impair lysosomal V-ATPase activity, leading to defective lysosomal acidification, reduced protease function, and accumulation of hyperphosphorylated tau, thereby accelerating AD progression (76).

### 5.5 Insulin signaling

Insulin signaling plays an essential role in regulating glucose metabolism, neuronal survival, synaptic function, and cognitive processes, primarily through the PI3K/Akt and Ras/Raf/MAPK pathways. In AD, impaired insulin signaling is associated with reduced IRS-mediated PI3K activity and decreased Akt phosphorylation, leading to enhanced activity of glycogen synthase kinase-3 (GSK-3), which promotes tau hyperphosphorylation and Aβ accumulation (77, 78). Akt downstream signaling through mTORC1 further connects insulin resistance with disrupted autophagy, altered synaptic plasticity, and pathological protein aggregation that contribute to memory decline in AD. Experimental studies have shown that targeting abnormal mTORC1 activation can restore autophagy and reduce Aβ and tau pathology, although conflicting findings regarding mTORC1 activity in AD still exist (79, 80). Additionally, inflammatory cytokines such as TNF-α and stress kinases including JNK, PKR, and IKK can induce inhibitory phosphorylation of IRS-1, thereby worsening brain insulin resistance and accelerating neurodegenerative dysfunction (81).

### 5.6 BBB dysfunction

The BBB, a key component of the neurovascular unit, is formed by endothelial cells, astrocytes, pericytes, and the basement membrane, and plays a vital role in regulating substance exchange, cerebral blood flow, and maintaining central nervous system homeostasis (82, 83). In AD, BBB dysfunction is characterized by impaired barrier integrity, altered transport mechanisms, reduced cerebral perfusion, and increased neuroinflammation. Damage to tight junction proteins, enhanced matrix metalloproteinase activity, and loss of pericytes contribute to BBB leakage, allowing toxic blood-derived molecules to enter the brain and trigger oxidative stress and inflammatory responses (84, 85). BBB disruption may also promote ischemic and hypoxic injury, further increasing Aβ production (86). Altered expression of BBB transporters is another major contributor to AD pathology, where reduced levels of LRP1 and impaired P-glycoprotein activity decrease Aβ clearance from the brain, while elevated RAGE expression enhances Aβ influx from the bloodstream into brain tissue (87, 88). Additionally, reduced expression of the glucose transporter GLUT-1 limits glucose uptake and energy metabolism in the brain, aggravating cerebrovascular dysfunction, amyloid pathology, neuronal degeneration, and cognitive decline (89).

*Peripheral immune–brain crosstalk in AD:* Although AD has traditionally been considered a disorder driven by central nervous system pathology, growing evidence indicates that bidirectional communication between the peripheral immune system and the brain significantly influences disease progression. Systemic inflammation can disrupt BBB integrity, facilitate the infiltration of peripheral immune cells and circulate inflammatory mediators into the brain parenchyma. BBB dysfunction, which occurs early during AD progression, enhances vascular permeability and promotes the entry of monocytes, neutrophils, and adaptive immune cells, thereby amplifying neuroinflammatory responses and contributing to neuronal dysfunction (90–92).

Among peripheral immune cells, circulating monocytes are recruited to the CNS through chemokine signaling pathways, where they may differentiate into macrophage-like cells. Experimental studies suggest that infiltrating monocytes can exert dual functions by facilitating Aβ clearance while also producing pro-inflammatory cytokines that exacerbate chronic neuroinflammation depending on the disease stage and local microenvironment. Similarly, neutrophils have been implicated in AD pathogenesis through increased adhesion to the cerebral vasculature, release of ROS, neutrophil extracellular traps (NETs), and inflammatory proteases, which collectively promote BBB damage, vascular dysfunction, and neuronal injury. However, much of the mechanistic evidence supporting neutrophil-mediated pathology has been derived from transgenic mouse models, while validation in human patients remains limited (92–94). A recent study shows that humans also support a potential role of neutrophils in AD pathology. In this study, microproteomics of human AD brain samples identified enrichment of immune-related pathways, including neutrophil extracellular trap formation, within Aβ plaques which shows the increased myeloperoxidase (MPO) levels and presence of MPO-positive neutrophils in brain capillaries and parenchyma of AD brains (95).

Adaptive immune responses also contribute to peripheral immune–brain crosstalk. Both CD4^+^ and CD8^+^ T cells have been detected in post-mortem AD brains and experimental models, suggesting that T-cell trafficking into the CNS may influence disease progression. Activated T-helper cells can enhance microglial activation and cytokine production, whereas Tregs may suppress excessive neuroinflammation and promote immune homeostasis. Nevertheless, the precise contributions of distinct T-cell subsets remain incompletely understood and are likely to vary across disease stages and patient populations (96–99).

Collectively, these findings support the concept that peripheral immune responses interact dynamically with central neuroinflammation rather than acting as independent drivers of AD pathology. While accumulating evidence from animal models, post-mortem studies, and observational human cohorts highlights the importance of immune–brain crosstalk, further longitudinal and interventional clinical studies are required to establish causal mechanisms and determine whether targeting peripheral immune pathways can modify disease progression. A recent study shows that human evidence also supports a potential role of neutrophils in AD pathology. A 2025 microproteomics study of human AD brain samples identified enrichment of immune-related pathways, including neutrophil extracellular trap formation, within Aβ plaques. The study also reported increased myeloperoxidase (MPO) levels and the presence of MPO-positive neutrophils in brain capillaries and parenchyma of AD brains. These findings provide human evidence that supports the neutrophil-associated inflammatory responses observed in experimental mouse models.

## 6 Biomarkers in AD diagnosis

The Institute, known for its excellence, named as The National Institute on Aging and Alzheimer’s Association (NIA-AA), developed research outlines, known as the AT(N) framework which defines the AD natural science by using biomarkers that are associated with Aβ accumulation, pathological tau, and neurodegeneration (100). These biomarkers are blood-based markers, cerebrospinal fluid (CSF) and imaging. Each of them plays an important role in the diagnosing and treatment of AD (101).

### 6.1 Imaging biomarkers

Advanced imaging tools like magnetic resonance imaging (MRI) and positron emission tomography (PET) are recently used at high level to study the brain’s structure and function in living individuals for checking real world scenario. Structural MRI (sMRI) helps to identify the shrinkage in the areas like hippocampus and entorhinal cortex, which are part of the medial temporal lobe. On the other hand, 18-fluoro-deoxy-glucose (18FDG)-PET measures how well the brain uses glucose, often showing reduced activity in regions like the posterior cingulate and temporoparietal areas (102, 103). PET scans can also detect the accumulation of the Aβ plaques and tau tangles, key markers of AD. sMRI and 18FDG-PET are effective in detecting neurodegenerative changes but these imaging findings are not specific to AD. These changes may also be observed in other neurological disorders (104). These methods can’t easily distinguish AD from other conditions like frontotemporal dementia or TDP-43-related disorders like proteinopathies. Also, atypical forms of AD or presence of cerebrovascular diseases can make diagnosis even trickier (105–107). Because of these challenges, imaging results are typically combined with clinical evaluations to improve the accuracy of diagnosis and treatment.

The specialized imaging techniques, like Aβ PET scanning and tau PET scanning, provide greater precision. Aβ PET scanning detects the buildup of Aβ plaques, while tau PET highlights tau tangles, both of which are markers of AD and can appear earlier than signs of brain degeneration (107). Various Research shows that amyloid PET is about 90% accurate for diagnosing AD and tau PET differentiates AD from other neurodegenerative diseases, outperforming traditional MRI markers. These advanced tools help so many doctors worldwide to detect AD-related changes earlier and with greater confidence (102).

Although amyloid- and tau-PET substantially improve diagnostic confidence, they have important limitations that restrict their widespread clinical application. Amyloid PET frequently detects cerebral Aβ deposition in cognitively normal older adults, thereby limiting its specificity for symptomatic Alzheimer’s disease. Conversely, a negative amyloid PET scan effectively excludes AD as the primary cause of cognitive impairment, resulting in excellent negative predictive value. Tau PET demonstrates greater disease specificity because tau burden correlates more closely with neurodegeneration and cognitive decline. However, off-target binding of first-generation tau tracers, limited sensitivity during the earliest disease stages, high imaging costs, radiation exposure, and restricted availability continue to limit routine clinical use (108, 109). Similarly, structural MRI and FDG-PET remain valuable for assessing neurodegeneration and excluding alternative neurological disorders but lack sufficient sensitivity and specificity to independently establish an AD diagnosis. Therefore, current international diagnostic guidelines recommend integrating imaging biomarkers with fluid biomarkers and comprehensive clinical assessment to maximize diagnostic accuracy (110–112).

### 6.2 CSF biomarkers

The framework of AT(N) uses several CSF biomarkers for example phosphorylated tau (P-tau), Aβ42 (Aβ42/Aβ40 ratio), and total tau (T-tau). All of these, P-tau181 is especially good at telling differences between AD and other dementias (113, 114). Amyloid PET scanning, tau PET imaging and CSF biomarkers help us to understand the progression of AD, but they aren’t exactly the same. For example, amyloid PET and CSF Aβ levels show a strong negative link, while CSF P-tau and tau PET results don’t always align. This difference occurs because CSF biomarkers show soluble tau levels, whereas tau PET captures insoluble tau deposits, which are more closely linked to decline in cognitive behavior. Recent studies show that CSF biomarkers like P-tau181, Aβ42, neurofilament light chain (NfL) and T-tau can reveal changes related to AD approximately 10–20 years before clinical symptoms clinically. This proposes that CSF biomarkers might be better at detecting the progression of the disease earlier than imaging techniques and give better option for treatment (23, 115).

Although CSF biomarkers remain the reference standard for detecting AD-related pathology, several limitations restrict their widespread clinical implementation. Measurement of CSF Aβ42/Aβ40, phosphorylated tau (particularly p-tau181 and p-tau217), and total tau demonstrates high diagnostic accuracy for distinguishing AD from cognitively normal individuals and many non-AD dementias (116). However, lumbar puncture is an invasive procedure that may reduce patient acceptance and is less suitable for repeated longitudinal monitoring. Furthermore, pre-analytical variables, including sample collection, storage conditions, tube adsorption, and inter-laboratory assay variability, can influence biomarker measurements despite ongoing international standardization efforts. While CSF p-tau biomarkers exhibit excellent sensitivity and specificity for AD pathology, total tau primarily reflects neuronal injury and is not disease-specific, as elevated concentrations are also observed in other neurodegenerative disorders, acute stroke, traumatic brain injury, and prion diseases (113, 116). Therefore, interpretation of CSF biomarkers should always be integrated with clinical evaluation, neuroimaging findings, and complementary biomarker data to improve diagnostic confidence.

### 6.3 Emerging CSF biomarkers

In addition to the AT(N) framework, researchers are investigating new CSF biomarkers to better understand different aspects of AD. The framework includes various markers that reflect damage to nerve Fibers (such as neurogranin and NfL), problems with communication between brain cells (like visinin-like protein 1, synaptosomal-associated protein 25), inflammation in the brain (including YKL-40, TREM2 and GFAP) and changes in brain metabolism (such as ceramide, sphingomyelin) (107, 117, 118). Another promising area of study involves very small particles in the CSF called extracellular vehicles (EVs) which contain proteins like CatB and C1q that could serve as early indicators of AD. These advancements aim to provide a more comprehensive picture of the disease and improve early detection and treatment (119, 120).

Emerging CSF biomarkers provide important insights into synaptic dysfunction, neuroinflammation, lipid metabolism, and extracellular vesicle biology, offering opportunities to characterize disease mechanisms beyond the classical AT(N) framework. However, most of these biomarkers remain at the discovery or early validation stage, and their clinical utility has yet to be fully established (121, 122). Many candidate biomarkers, including neurogranin, YKL-40, soluble TREM2, synaptosomal-associated protein-25 (SNAP-25), visinin-like protein-1 (VILIP-1), and extracellular vesicle-associated proteins, demonstrate promising associations with disease progression but exhibit variable diagnostic performance across independent cohorts. Neuroinflammatory biomarkers provide valuable information about immune changes occurring AD progression and can complement traditional amyloid and tau biomarkers. GFAP reflects astrocyte activation and increases early in AD, making it a potential marker for early disease detection. Soluble TREM2 (sTREM2) indicates microglial activation and helps monitor changes in microglial function during disease progression. YKL-40 and inflammatory cytokines such as IL-1β, IL-6, and TNF-α represent ongoing glial activation and chronic inflammation associated with neuronal damage. These biomarkers may help identify patients with active neuroinflammation and evaluate responses to anti-inflammatory therapies; however, their clinical use is still limited due to differences in disease stage, biomarker variability, and the need for further validation. In addition, the absence of standardized analytical platforms, universally accepted diagnostic cut-off values, and large multicentre validation studies currently limits their incorporation into routine clinical practice (116). Future investigations integrating these biomarkers with established AT(N) markers and blood-based biomarkers may improve patient stratification, enable earlier detection of disease progression, and facilitate personalized therapeutic interventions.

### 6.4 Blood biomarkers

Biomarkers based on blood provide a less invasive and more accessible way to detect AD compared to CSF and imaging methods. Developments in highly sensitive analytical platforms have made it possible to detect AD-related proteins like Aβ, NfL, P-tau and GFAP in blood samples (24, 123). For instance, a recent test measured 21 AD-related blood biomarkers, revealing information about neurodegeneration, vascular function, inflammation and metabolic activity. Using this data, machine learning models have shown high accuracy in identifying AD, Aβ pathology and MCI in diverse populations (124).

Blood-based biomarkers have emerged as attractive first-line screening tools because they are minimally invasive, relatively inexpensive, and suitable for repeated longitudinal monitoring compared with CSF analysis and PET imaging. Plasma phosphorylated tau isoforms (particularly p-tau181, p-tau217, and p-tau231) have demonstrated excellent diagnostic performance for detecting AD pathology, with p-tau217 consistently showing the highest diagnostic accuracy for predicting amyloid and tau positivity in numerous independent cohorts (125, 126). However, diagnostic performance varies according to assay platform, disease stage, analytical methodology, and patient comorbidities. Plasma Aβ42/Aβ40 ratios generally exhibit lower sensitivity than phosphorylated tau biomarkers and may be influenced by peripheral Aβ production. Likewise, plasma GFAP and neurofilament light chain are highly sensitive indicators of astrocytic activation and neuroaxonal injury, respectively, but lack disease specificity because their concentrations are also elevated in other neurodegenerative disorders, traumatic brain injury, multiple sclerosis, stroke, chronic kidney disease, and systemic inflammatory conditions. Consequently, no single plasma biomarker is currently sufficient for definitive diagnosis, reinforcing the need for multi-marker panels combined with clinical evaluation (127–129).

From a health-economic perspective, plasma biomarkers offer substantial cost-effectiveness compared with amyloid PET and CSF testing. Their relatively low cost, scalability, and compatibility with primary care settings make them well-suited for population-level screening and identifying individuals who should undergo confirmatory PET imaging or CSF testing. Recent modeling studies suggest that blood-based biomarker-guided diagnostic pathways could substantially reduce healthcare costs while improving access to disease-modifying therapies through earlier diagnosis and more efficient patient selection.

The regulatory landscape for plasma biomarkers is also evolving rapidly. Several commercially available laboratory-developed tests measuring plasma p-tau217, p-tau181, Aβ42/Aβ40, and GFAP are already implemented in specialized clinical laboratories (129). Importantly, the US-FDA recently authorized the Lumipulse^®^ G pTau217/β-Amyloid 1–42 Plasma Ratio test as an *in vitro* diagnostic aid for detecting amyloid pathology in adults with cognitive impairment undergoing evaluation for AD, representing a major milestone toward routine clinical implementation of blood-based biomarkers. Nevertheless, additional multicentre validation, assay standardization, establishment of universal reference ranges, and harmonization across analytical platforms remain essential before widespread global clinical adoption (130, 131).

Although blood-based biomarkers have substantially improved the diagnosis, prognosis, and therapeutic monitoring of AD, several challenges remain before their widespread clinical implementation. Biomarker levels may be influenced by coexisting conditions, including vascular diseases such as hypertension, chronic kidney disease, traumatic brain injury, and other neurodegenerative disorders (e.g., α-synucleinopathies), potentially reducing diagnostic specificity. Therefore, future research should focus on improving biomarker specificity and validating their performance across diverse patient populations and clinical settings. Furthermore, combining AT(N) biomarkers with non-AT(N) biomarkers, including markers of neuroinflammation, synaptic dysfunction, and neurodegeneration, has significantly enhanced diagnostic accuracy and disease characterization (132). Composite biomarker panels integrating plasma biomarkers, cerebrospinal fluid biomarkers, neuroimaging findings, genetic risk factors, and clinical data offer a more comprehensive assessment of AD, enabling earlier diagnosis, improved patient stratification, monitoring of disease progression, and evaluation of therapeutic response. Continued efforts toward assay standardization, large-scale clinical validation, and integration of multimodal biomarkers into routine clinical practice will be essential for realizing precision medicine in AD (133–135).

## 7 Clinical trials and the mechanism of action of drugs in AD treatment

AChEIs and NMDA receptor antagonists are the two kinds of traditionally used AD medications. Memantine is classified as an NMDA receptor antagonist, while tacrine, donepezil, rivastigmine, and galantamine are classified as AChEIs. By inhibiting acetylcholinesterase and thereby increase cholinergic signaling, AChEIs promote postsynaptic activity, improving patients’ cognitive and behavior abilities (136). Tacrine was first made available for the treatment of AD in 1993. However, it was taken off the market due to its high liver toxicity. Despite this, tacrine shows promise in the study of multitarget-directed ligands (137, 138). Galantamine, rivastigmine, and donepezil are more selective second-generation AChEIs. Second-generation AChEIs are first-line medications for AD because they had improved pharmacokinetic characteristics or revealed fewer adverse effects. To minimize adverse effects and improve patient adherence, current research is focused on optimizing dosage forms, administration techniques, and combinations for these widely used drugs (139). The FDA authorized Adlarity, a donepezil transdermal patch, in 2022 for the treatment of AD at all stages. Adlarity is administered once a week, which has the same effectiveness as the daily oral dose but causes fewer gastrointestinal problems. In addition, it is more convenient than the once-daily rivastigmine patch (140, 141) (**Figure 4**).

**Figure 4:**
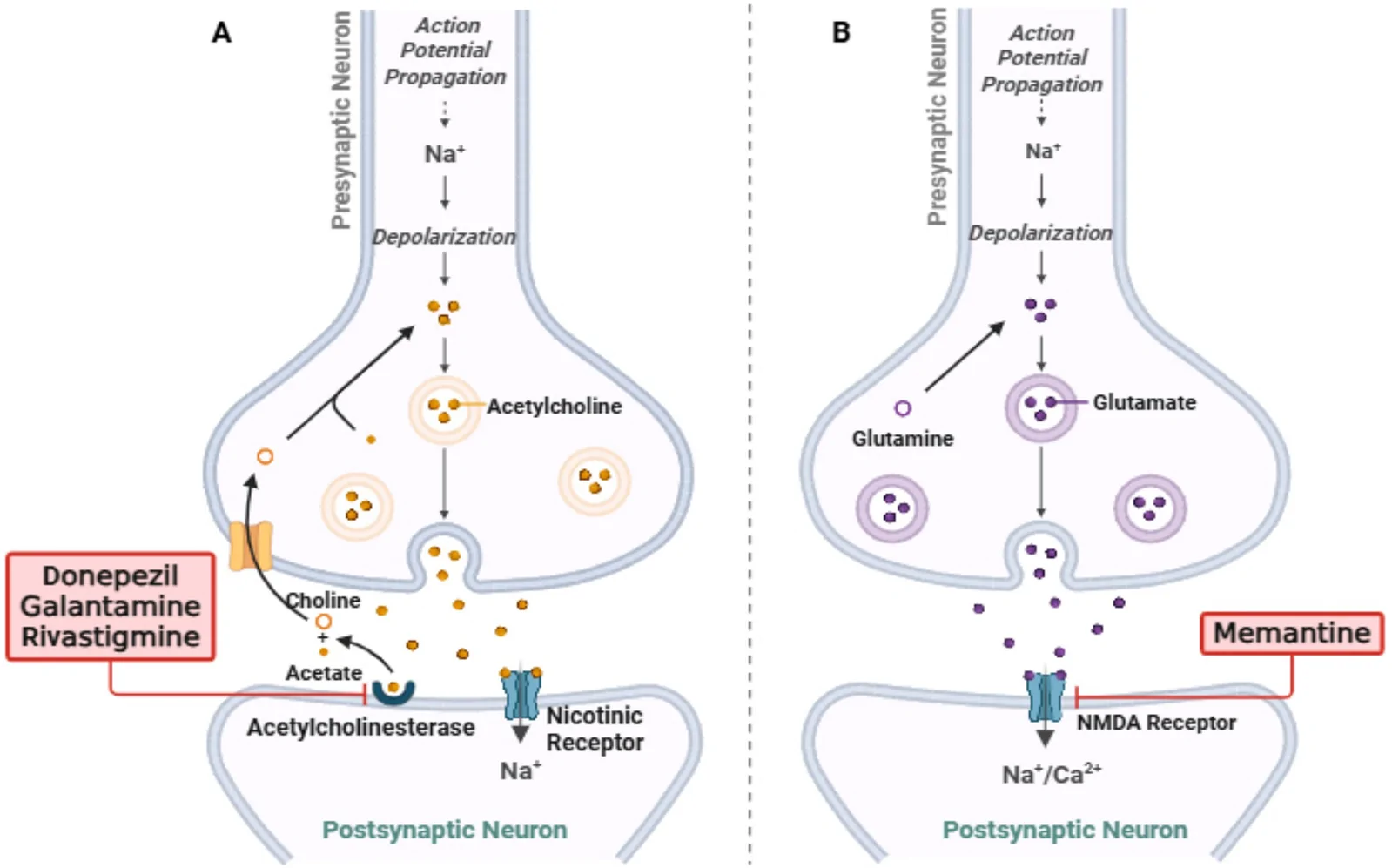
Mechanism of action of anti-acetylcholinesterase (AChEIs) and NMDA receptor inhibitors. Image illustrates the mode of action of **(A)** AChEIs (Donepezil, Galantamine, and Rivastigmine) and **(B)** NMDA receptor inhibitors (Memantine).

Cholinesterase inhibitors are also being delivered using nanocarriers via intranasal, intravenous, and other routes. Nanocarriers play a crucial role in improving medication concentration, release, and bioavailability (142, 143). Unexpected advantages can be obtained while treating AD by using the right mixtures of cholinesterase inhibitors, such as donepezil and galantamine, or by combining them with other neurological drugs, metal chelators, or antioxidants. Safety, tolerance, and efficacy will all increase as a result (144, 145). Memantine, an FDA-approved NMDA receptor antagonist, which is responsible for memory is effective for moderate to severe AD. It helps patients’ behavior, everyday life skills, and cognitive function by acting as NMDA receptor antagonist (146, 147). For moderate to severe AD, Namzaric, a fixed-dose combination of donepezil and memantine extended-release, provides an additional choice. These medications primarily alter neurotransmitter levels; however, they do not alter the progression of the disease. They offer insightful information for creating novel therapies (148).

The idea of “disease-modifying therapy for AD” was put up by researchers in 2017 to influence underlying biological processes to stop the disease’s progression and offer patients long-term advantages (149, 150). Among these, Sodium oligomannate (GV-971), an oligosaccharide derived from marine algae, received conditional approval in China in 2019 for the treatment of mild-to-moderate AD. However, its approval has been met with considerable international scepticism because the reported cognitive benefits, although statistically significant, have been questioned regarding their clinical relevance, and independent validation from large multinational clinical trials remains limited. The proposed mechanism of action involves modulation of the gut microbiota, leading to reduced peripheral and central neuroinflammation, in addition to inhibiting Aβ aggregation. Nevertheless, this gut microbiota–neuroinflammation axis remains a proposed mechanism and continues to be actively debated (151, 152). Subsequent preclinical studies revealed that in both the APPPS1–21 and 5xFAD models, GV-971 altered the gut microbiome’s abundance and composition in a sex-dependent manner. This alteration affected peripheral inflammation and microbial metabolism, controlled microglia activation and activity, and eventually decreased amyloidosis and neuroinflammation (153). To further investigate the efficacy and safety of certain treatments, phase IV clinical trials for NCT05181475 and NCT05058040 have recently begun, with results anticipated to last until 2026. Although these findings support the therapeutic potential of GV-971 in experimental models, their translation to clinical benefit in humans remains to be conclusively established. Further large-scale, independent clinical studies are therefore required to validate its efficacy, clinical utility, and proposed mechanism of action.

There are three monoclonal antibodies that targeting amyloid-β (Aβ) e.g. Aducanumab, Lecanemab, and Donanemab which played a crucial role in the development of disease-modifying therapies for AD. Although several earlier Aβ-targeting therapies failed to demonstrate clear clinical benefits, recent advances shown that targeting specific forms of Aβ can provide modest clinical benefits in selected patients with early AD (154, 155). Lecanemab received traditional FDA approval in 2023, while Donanemab (Kisunla) received FDA approval in July 2024 for patients with mild cognitive impairment or mild dementia due to AD (156). In contrast, Aducanumab received accelerated FDA approval in 2021 (157). but its clinical use was limited because of uncertainty surrounding its clinical benefit, concerns regarding the interpretation of the EMERGE and ENGAGE trials, and its high risk of amyloid-related imaging abnormalities (ARIA), including cerebral edema and microhaemorrhages. Aducanumab was subsequently withdrawn from the U.S. market in 2024. These antibodies differ in their Aβ-binding profiles: Aducanumab recognizes aggregated soluble oligomers and insoluble fibrils (158), Lecanemab preferentially binds soluble Aβ protofibrils, and Donanemab selectively recognizes N-terminal pyroglutamate-modified Aβ (pGlu3-Aβ), which is enriched in established amyloid plaques and promotes plaque removal. Clinical studies of Lecanemab and Donanemab have demonstrated substantial reductions in cerebral amyloid burden and modest slowing of cognitive decline in carefully selected patients with early AD. Treatment generally requires confirmation of amyloid pathology using amyloid PET imaging or cerebrospinal fluid biomarkers. However, these therapies are associated with ARIA and therefore require regular MRI monitoring, particularly in patients carrying the APOE ϵ4 allele. Repeated intravenous infusions, biomarker testing, safety monitoring, and high treatment costs also remain important barriers to their widespread clinical use. Thus, the development of Aβ-targeting antibodies has evolved from repeated clinical failures toward more selective therapies that have demonstrated measurable, although modest, clinical benefits in appropriately selected patients (159, 160). Brexpiprazole represents a distinct symptomatic therapeutic approach rather than a disease-modifying anti-amyloid strategy. It is an atypical antipsychotic that acts as a partial agonist at dopamine D_2_ and serotonin 5-HT_1_A receptors and an antagonist at serotonin 5-HT_2_A receptors. Brexpiprazole has been approved for the treatment of agitation associated with Alzheimer’s dementia and has demonstrated efficacy in reducing agitation symptoms without targeting the underlying amyloid pathology (161, 162).

Current therapies for AD are investigating the fundamental underpinnings of the disorder, revealing several potential therapeutic targets. These advancements hold potential for slowing or perhaps stopping the growth of AD. To fully comprehend how these drugs function, assess their long-term utility, and confirm their safety, more research is needed. Given the challenging risk-benefit ratio in the treatment of AD, repurposing existing drugs has become standard procedure. The costly, time-consuming, and resource-intensive process of developing new AD strategies has a high failure rate and has sparked interest in modifying medications initially meant for other conditions, including diabetes, cancer, heart disease, psychiatric disorders, and different neurological disorders. In addition to having the potential for several medical uses, these repurposed medications frequently have well-understood and well-executed effectiveness and safety profiles (163–165). Additionally, the growing number of computational tools powered by AI is improving the impacts of drug repurposing, providing a viable path for AD drug discovery (166, 167). According to data from ClinicalTrials.gov, 187 clinical trials focusing on AD and MCI have recently been conducted, spanning across stages I, II, and III. 36 medications are in phase III, 87 are in phase II, and 31 are in phase I of these trials (168).

NMDA receptors, adrenoceptors, AChE, nicotinic α7 receptors, and 5-hydroxytryptamine receptors are among the targets of the principal pharmacological activity in this specific setting (169, 170). In this case, γ-secretase inhibitors or modulators, α-secretase activators, and BACE1 inhibitors are used to reduce Aβ. There are further tactics that concentrate on preventing Aβ buildup or enhancing its removal with certain vaccinations and antibodies. It can stabilize the microtubules and prevent tau protein aggregation by modifying tau protein phosphorylation. (**Figure 5**) Alternatively, we can utilize antibodies and vaccines to stabilize the microtubules. with the use of certain modulators and anti-inflammatory medications (NSAIDs) to increase microglia activity. Neurotransmitter receptors and Aβ-related pathways are the focus of many phase-II and III trials, whilst phase II trials are being conducted for medications that reduce neuroinflammation and tau aggregation, including repurposed medicines (170, 171).

**Figure 5:**
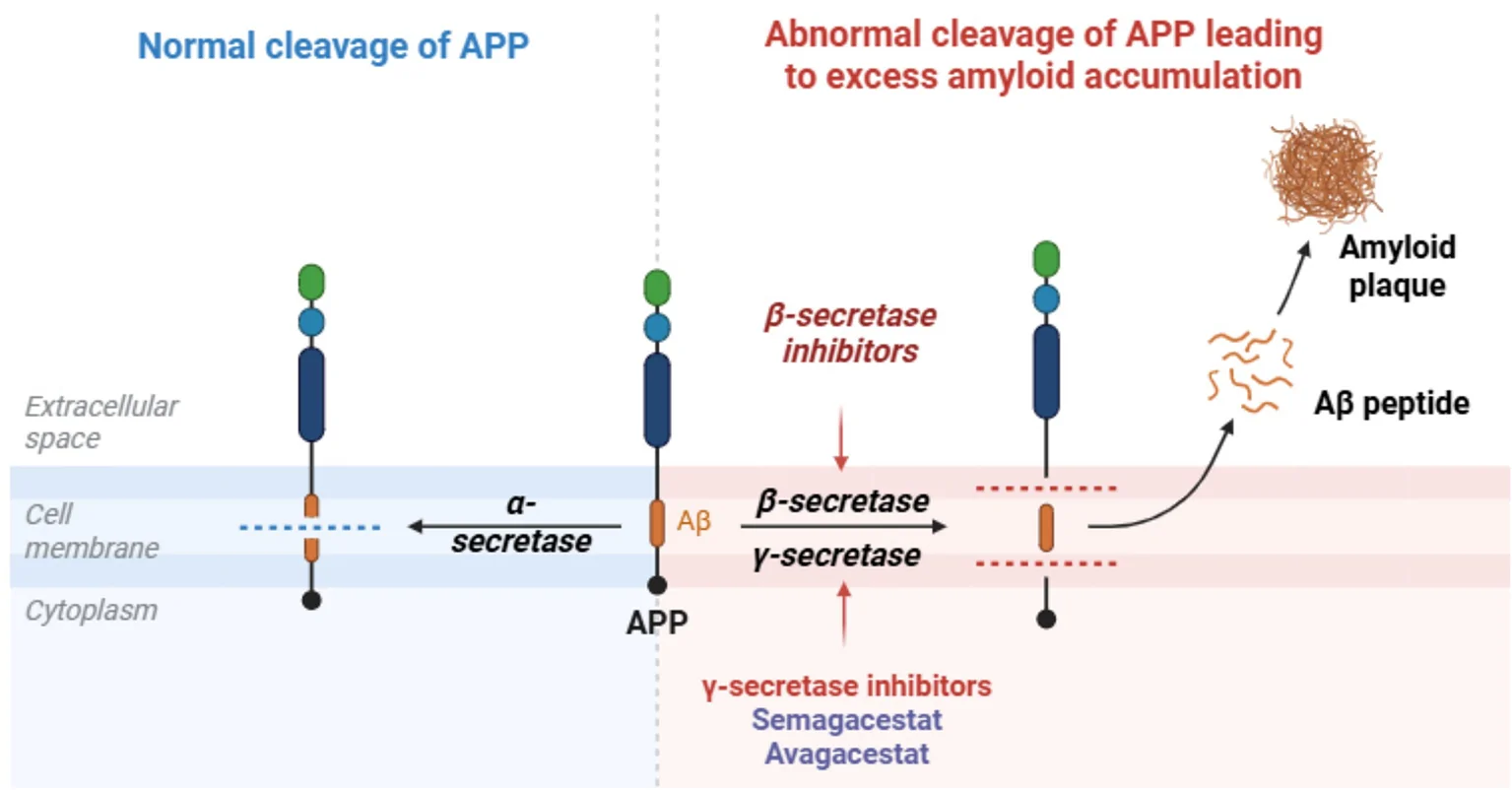
The mechanism shows the activity of α/β/γ-secretase in APP cleavage. Figure illustrates the involvement of (*Left side panel*) α-secretase in the normal cleavage of APP, and (*Right side panel*) shows the role of β/γ-secretase in the abnormal cleavage of APP, which leads to the formation of Aβ plaque. However, this figure also shows the inhibition of β/γ-secretase to control its abnormal cleavage.

**Table 2** lists some of the important medications for AD that have moved to the clinical stage. Of these, the medication undergoing phase III trials as the first γ-secretase inhibitor is Semagacestat (LY-450139). However, a long-term clinical trial (NCT005945) suggested that tracking the progression of AD revealed surprising findings. Every participant in every group exhibits a decline in their cognitive and functional abilities. In addition, there were other adverse effects from the trial, such as gastrointestinal problems, skin cancer, and other infections. Researchers believe that these side effects may be caused by the drug’s impact on γ-secretase substrates, which include CD44, ErbB4, notch, and cadherin (172, 173). Another well-known medication, Avagacestat (BMS-708163), exhibits remarkable activity for APP-C99 as an oral γ-secretase inhibitor when compared to Semagacestat. Phase I trials demonstrate its potential as a reduction in the activity of Aβ plaque accumulation, one of the major causes of AD. The group with larger doses displayed side effects similar to those reported with semagacestat, including gastrointestinal problems and skin cancer, even though phase II trials (NCT00890890) evaluate the safety and tolerability in people with primary stage AD. These findings highlight the challenges in developing safe and efficient therapies for AD (174, 175).

**Table 2:** List of drugs in clinical trials for AD treatment and their mechanism of action. (source: https://clinicaltrials.gov).

S. no	Drug candidate	Target	Company	NCT No.	Phase	Status/stage	Outcome/safety
γ-secretase inhibitor							
1	Semagacestat	γ-secretase	Eli Lilly	NCT00594568	III	C	Improvement in orientation, verbal memory, language, and praxis
2	Avagacestat	γ-secretase	Bristol-Myers Squibb	NCT00890890	II	T	No promising results and safety.
BACE1 reversible inhibition							
3	Umibecestat	BACE1	Novartis	NCT02565511 NCT03131453	II/III II/III	T T	No promising results and safety.
4	Elenbecestat	BACE1	Eisai Co., Ltd	NCT02956486	III	T	No promising results and safety.
Prevent oligomer formation							
5	ALZ-801	Oligomer formation	Alzheon Inc.	NCT04770220	III	A, NR	Ongoing.
Glutaminyl cyclase inhibitor							
6	Varoglutamstat	Glutaminyl cyclase	Vivoryon Therapeutics N. V	NCT03919162 NCT04498650	II II	A, NR C	Ongoing. Improvement in symptoms.
Prevent Tau aggregation							
7	Tideglusib	Tau	Noscira SA	NCT01350362	II	C	–
8	TRx0237	Tau	TauRx Therapeutics Ltd	NCT01689233 NCT01689246 NCT03446001	III III III	C C C	–
Target inflammation & neuro-inflammatory system							
9	NE3107	Inflammation	BioVie Inc.	NCT04669028	III	C	–
10	ALZT-OP1	Inflammation	AZTherapies, Inc.	NCT02547818	III	C	–
11	Masitinib	Inflammation	AB Science	NCT05564169	III	NR	Ongoing.
Calcium channel blocker							
12	Nilvadipine	Ca^2+^ channel	Prof Brian Lawlor	NCT02017340	III	C	–
PPARγ agonist							
13	Pioglitazone	PPARγ	Takeda	NCT01931566 NCT02284906	III III	T T	No promising results and safety
Anti-amyloid monoclonal antibody							
14	Solanezumab	Aβ	Eli Lilly	NCT00905372 NCT00904683 NCT01900665	III III III	C C T	–
15	Gantenerumab	Aβ	Hoffmann-La Roche	NCT03444870 NCT03443973	III III	T T	No promising results and safety
Anti-tau monoclonal antibody/vaccine							
16	Bepranemab	Tau	UCB Biopharma SRL	NCT04867616	II	A, NR	Ongoing.
17	E2814	Tau	Washington University School of Medicine	NCT01760005 NCT05269394	II/III II/III	R A, NR	Ongoing.
18	AADvac1	Tau	Axon Neuroscience SE	NCT02579252	II	C	–
Anti-TREM2 monoclonal antibody							
19	AL002	TREM2	Alector Inc.	NCT04592874	II	A, NR	Ongoing.

PPARγ, Peroxisome Proliferator-Activated Receptor-γ; TREM2, Triggering Receptor Expressed on Myeloid Cells 2; A*β*, Amyloid-*β*; A: Active; C, Completed; R, Recruiting; NR, Not recruiting; and T, Terminated. These drugs were validated using multi-omics approaches.

In contrast to γ-secretase inhibitors, researchers looked into BACE1 inhibitors, which target β-secretase, as a more selective way to address APP by lowering Aβ synthesis and accumulation (176). Among these, Umibecestat, sometimes referred to as CNP520, has demonstrated remarkable safety and effectiveness from the initiation of clinical trials (177, 178). However, Phase II/III trials that included older persons with APOE4 allele carriers—who do not have cognitive symptoms but are identified as having an elevated risk of AD—were completed early. This decision was influenced by the participants’ privileges of mild cognitive deterioration and brain atrophy (179).

The fourth-generation BACE1 inhibitor Elenbecestat (E2609) also underwent phase III studies before being withdrawn, regrettably (180). The purpose of the phase III trial is to assess its safety and effectiveness in individuals with early-stage AD who were immobilized because of a number of dangers. Indeed, this medication has numerous adverse effects, such as liver damage, weight loss, skin rashes, and nightmares, and it has no benefit in cognitive function (181, 182). On the other hand, ALZ-801, which contains the oral API tramiprosate, is effective against Aβ oligomers. Unlike plaque-specific drugs, it did not adhere to plaques, potentially lowering the risk of ARIA, a known issue with plaque-clearing therapy (183, 184). According to the drug’s phase II research results (NCT04693520), it can lower biomarker levels and may slow down the deterioration of memory and learning skills in early-stage AD patients with the APOE gene (APOE3/4 or APOE4/4 genotypes). In light of these excellent results, a phase III trial (NCT04770220) is currently underway to further assess the drug’s safety and effectiveness, particularly in homozygous patients with APOE4 and early AD. These discoveries are expected to persist until 2024, when further details regarding its practical advantages will be available (162).

Another noteworthy medication that has advanced to phase II clinical trials is Varoglutamstat, sometimes referred to as PQ912, which is a glutaminyl cyclase inhibitor. The pharmacological activity of the medication targets an enzyme located at the N-terminus of Aβ that converts glutamate to pyroglutamate, causing more dangerous Aβ plaques to form (185). The efficacy and safety profile of varoglutamstat, NCT03919162, were favorable in phase IIa trials. Additionally, it was well tolerated and demonstrated a reduction in working memory deterioration. NCT04498650 is undergoing a phase IIb trial to examine its long-term safety and tolerance. Additionally, it looks into the impacts of cognitive decline and how it affects AD biomarkers (186) (**Figure 6**).

**Figure 6:**
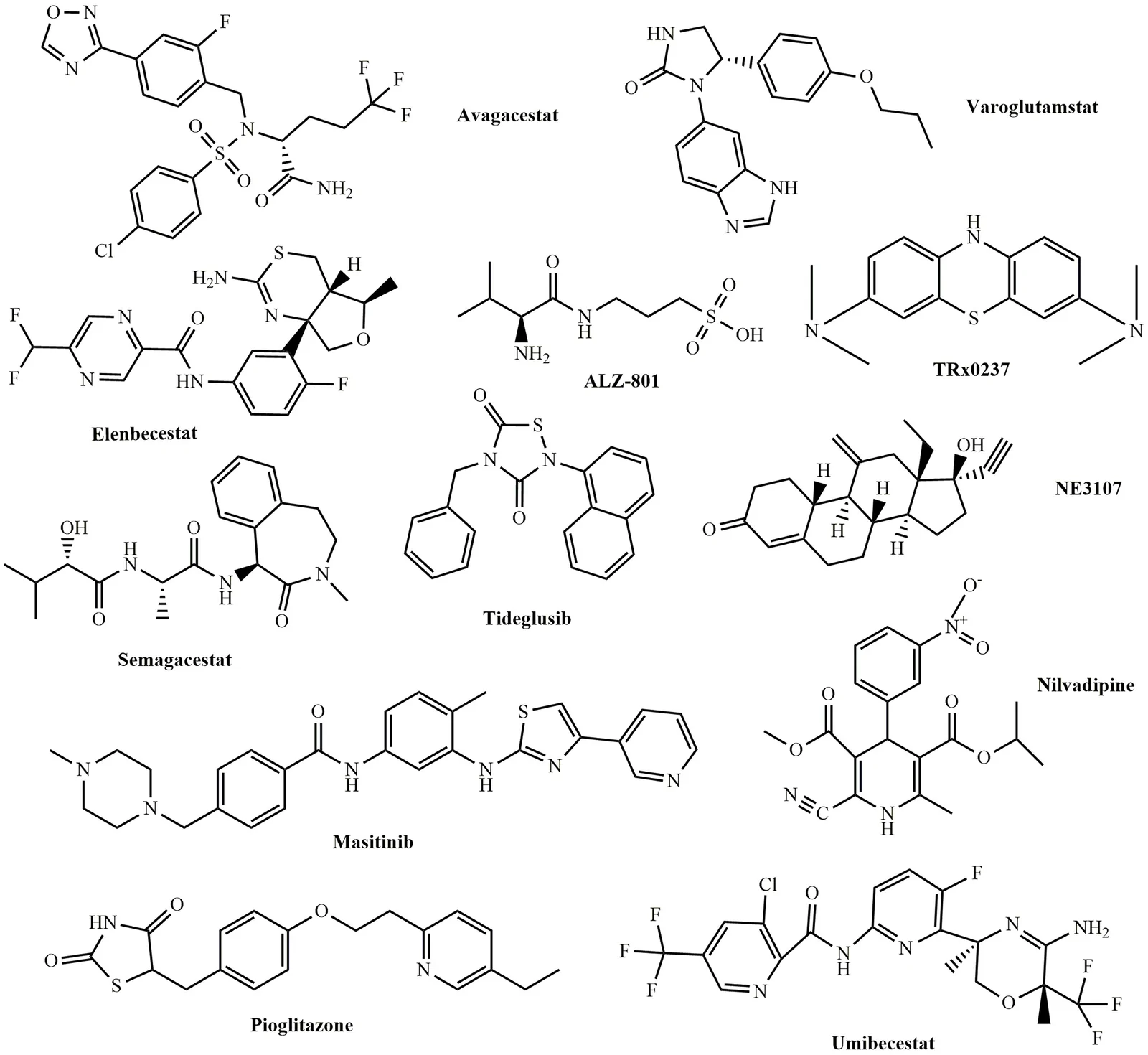
Chemical structures of the potential drugs in clinical trials. The image illustrates the structure of the drug molecules under clinical trials.

Another is Solanezumab (LY2062430), an antibody-based treatment that primarily targets the intermediate region of Aβ (186). Solanezumab primarily targets monomeric, soluble, and non-fibrillar forms of Aβ, representing a novel therapeutic approach aimed at disrupting amyloid plaque formation. Effective treatments for AD are being developed as a result of continuous discoveries. In two early phase III trials (NCT00905372 and NCT00904683), a medication’s effectiveness in treating mild to severe AD was assessed by comparing it to a placebo. The findings indicate that neither cognitive effects nor functional degradation were significantly reduced by the medication. Furthermore, there are a number of indications that it might change as the illness progresses in those with mild AD. Following the Phase 3 trial’s (NCT01900665) termination, the drug’s primary goals were lost when the validation of its effectiveness in patients with moderate AD was deemed ineffective in 2016 (187–189).

Gantenerumab, a subcutaneous antibody, was designed to engage with two specific areas of Aβ, the core structural domain and the N-terminal, which primarily target plaques, soluble oligomers, and protofibrils (190). Nevertheless, two phase III trials (NCT03444870 and NCT03443973) were stopped for use in early AD. Due to the drug’s limited ability to remove amyloid plaque, it showed little clinical benefit in decreasing cognitive deterioration. Furthermore, amyloid-related imaging abnormalities with effusion (ARIA-E) are among the negative effects that only 5% of individuals exhibit (190, 191).

Tideglusib is a non-ATP competitive inhibitor of GSK-3β that exhibits neuroprotective and anti-inflammatory effects. Additionally, phase-II research (NCT01350362) was conducted to evaluate the drug’s safety, effectiveness, and tolerability in patients with mild to moderate AD. The primary and secondary objectives were not achieved. In the meantime, TRx0237, sometimes referred to as LMTX, is an inhibitor of tau protein aggregation. Phase III trials have either been completed or terminated. NCT01689246 and NCT01689233 are two earlier studies that involved patients with mild and mild-to-moderate AD. According to the study, using the medication by itself was generally safe and showed promise (108, 192, 193). To further evaluate the safety and efficacy of a 16 mg/day dosage in comparison to a placebo in individuals with mild to moderate AD, a follow-up phase III trial (NCT03446001) is in underway; results have not yet been made public (194). Furthermore, the tau protein’s core region is the primary target of the antibody Bepranemab, also known as UCB0107, which demonstrates its ability to stop tau protein aggregation and spread. Its safety, tolerability, and efficacy in patients with moderate AD or mild cognitive impairment (MCI) are being assessed in a phase II trial (NCT04867616).

E2814 is another monoclonal antibody that inhibits the accumulation and spread of tau proteins by acting on the tau microtubule-binding region. Three clinical trials are now being conducted to test this medication (195). One is a phase I/II trial (NCT04971733) with results anticipated in 2025 that focuses primarily on safety, effectiveness, and tolerability as well as the capacity to engage its target in individuals with hereditary AD (DIAD). The effectiveness of E2814 + Lecanemab in patients with early-onset AD is being investigated in two more phase II/III trials (NCT01760005 and NCT05269394). These studies, which are expected to end in 2027, measure cognitive abilities whether or not they are altered, with tau PET imaging serving as their primary endpoint.

AADvac1 is a well-known tau vaccine that is currently undergoing clinical trials. It is specifically made to prevent tau aggregation, eliminate pre-existing tau proteins, and limit their dissemination, all of which contribute to a reduction in the rapid course of AD. To assess the safety and effectiveness in patients with mild AD, a phase II trial (NCT02579252) was conducted. It proved that the AADvac1 vaccine was well tolerated and has not yet been associated with any serious side effects. However, additional research is required to prove the clinical effectiveness (162, 196).

The small-molecule medication NE3107, commonly referred to as HE3286, targets insulin sensitizers and reduces inflammation. The phase III clinical trial NCT04669028 was mostly conducted for elderly people with mild to moderate AD and produced positive outcomes. Although NE3107 was not well accepted, it greatly improved cognitive functioning and slowed the loss in cognitive activity. In the meantime, ALZT-OP1 is a mix of ibuprofen and cromolyn sodium. By simultaneously targeting the Aβ and preventing their aggregation, the combination encourages the microglial cells to change into a pro-phagocytic state and exhibit neuroprotective effects. ALZT-OP1’s phase III research (NCT02547818) was finished with the goal of determining its efficacy and safety in people with early AD evaluation. The purpose of this experiment is to ascertain whether the medication can reverse cognitive decline in individuals who are in the early stages of the illness (197, 198).

TREM2, a protein implicated in microglial activation, is targeted and activated by the monoclonal antibody AL002. AL002 increases TREM2 activity in an attempt to reduce the development of Aβ plaque and neurite degeneration, both of which are linked to AD. The safety and effectiveness of AL002 in individuals with early-stage AD are currently being investigated in a phase II clinical trial (NCT04592874), which shows promise as a novel therapeutic approach (198). Masitinib, a highly selective tyrosine kinase inhibitor that targets the many pathways implicated in AD, is another intriguing possibility. It works by controlling signaling pathways linked to tau and Aβ proteins, preventing synaptic damage, and decreasing the activation of mast cells and microglia. A phase III trial (NCT05564169) will examine the possibility of mastinib as an adjuvant therapy for conventional treatments like cholinesterase and memantine inhibitors. The trial’s goal is to determine whether masitinib can improve cognitive and functional outcomes for patients with mild to moderate AD (199, 200).

Drug repurposing: Researchers are also investigating the possibility of repurposing drugs for the treatment of AD for a variety of novel drugs. For instance, Pioglitazone, a well-known and successful medication for diabetes, and nilvadipine, a calcium channel blocker that was formerly used to treat hypertension, are being researched for their possible benefits in AD. These initiatives demonstrate the innovative methods being used to treat this challenging and complex illness. Nilvadipine has various beneficial properties, including the capacity to improve cerebral blood flow, reduce the formation of Aβ, and exhibit anti-inflammatory and anti-tau actions (201, 202). A phase III trial (NCT02017340) was conducted to evaluate its efficacy and safety in individuals with mild to severe AD. This led to contradictory results in spite of these promising avenues. Despite being safe, the medication did not considerably slow down the cognitive loss of AD patients, which emphasizes how difficult it is to convert these qualities into quantifiable clinical advantages. A well-known PPARγ agonist used to treat type 2 diabetes (T2D), pioglitazone has been investigated for its promising potential in treating AD. The safety and effectiveness of AD-prepared phase III clinical studies (NCT01931566 and NCT02284906) in individuals with moderate cognitive impairment (MCI) were examined. However, due to the drug’s failure to demonstrate significant therapeutic benefit, both trials were ended. These results emphasize the difficulty of repurposing current drugs for the treatment of AD and the significance of continuing research into more specialized therapies (203–205).

Selection of appropriate drug targets: depending on biomarkers and animal models during research, and other complications like delayed treatment initiation, dose-related side effects, issues crossing the blood-brain barrier, and the disease’s varied manifestations in different people are just a few of the difficulties that have been faced in the research and development of medications for AD. According to the Aβ hypothesis, Aβ is a crucial therapeutic target (205, 206). The structural variability of Aβ, which comprises monomers, soluble oligomers, protofibrils, amyloid plaques, and pathogenic forms, makes it a significant obstacle. This affects the creation of medications and makes it very difficult to determine an exact therapeutic target. Although several early Aβ-targeting therapeutic strategies failed to demonstrate meaningful clinical benefit or raised significant safety concerns (such as bapineuzumab), however the recent approvals of Lecanemab and Donanemab have renewed interest in anti-amyloid therapy, demonstrating modest clinical benefit in carefully selected patients with early AD. Targeting the β- and γ-secretases, which are in charge of the creation of Aβ, produced detrimental effects because these enzymes are also crucial for other physiological functions. Perhaps impeding progress in the field, the focus on the theory has hindered research on alternative therapeutic targets. Furthermore, biomarkers have become essential for AD in terms of dosage optimization, biological effect detection, patient selection, and therapy progress tracking. Aβ monoclonal antibodies were recently approved, which has been substantially aided by the availability of more accurate biomarkers. The disparity between the effectiveness of drugs in preclinical studies and human trials has also led to improvements in animal models for AD. In an effort to better mimic the progression of AD, researchers are using multi-gene transgenic models, which account for species differences and non-genetic factors in addition to a single genetic mutation (207–210).

The most common treatment is still immunotherapy, which specifically targets the tau and Aβ proteins, but small-molecule medications are becoming more and more popular. These medications are good choices for treating both established and new therapeutic targets because of their adaptability, ease of use, and proven development techniques (169). Research on next-generation small-molecule treatments for AD is therefore quite interesting. The safety, effectiveness, and drug-like qualities of these treatments are also being enhanced by a number of novel inhibitory techniques, such as selective, dual-targeted, allosteric, covalent, PROTACs, and those that target protein-protein interactions (PPIs). In addition to showing potential for addressing a larger number of AD targets, this multifaceted approach aims to minimize common challenges and expedite drug development.

The limited success of first-generation Alzheimer’s disease therapies can be attributed to several factors rather than failure of a single therapeutic target. Many clinical trials enrolled patients with mild-to-moderate Alzheimer’s disease, a stage at which extensive neuronal loss and irreversible brain damage had already occurred, reducing the potential therapeutic benefit. In addition, several drugs showed poor BBB penetration, insufficient target engagement, or dose-limiting adverse effects that prevented effective treatment. Furthermore, therapies targeting only a single pathological process, such as amyloid-*β* or tau, failed to address the complex interactions among neuroinflammation, synaptic dysfunction, and neuronal degeneration. These limitations have shifted research toward next-generation therapies that emphasize earlier intervention, improved brain delivery, and multi-target approaches to achieve better clinical outcomes.

## 8 Potential next-generation inhibitors for AD treatment and management

AD is caused by a variety of conditions, and the intricate relationships between these factors make it particularly challenging to develop effective treatments. While many intriguing cures fail in clinical trials due to unfavorable side effects or lack of significant efficacy, conventional treatments have had little success. This has raised the standard for the next generation of AD medications, which aim to provide a range of targeted therapies based on each patient’s unique situation and disease progression. Advances in drug development techniques and our increasing understanding of the disease’s fundamental origins have opened up new pathways for the discovery of potential therapies. **Table 3** provides a summary of the next-generation medications being researched for AD.

**Table 3:** List of under-development next-generation compounds for AD treatment and management.

S. no	Drug candidate	Model (*In-vitro*/*In-vivo*)	Outcome
Targeting GSK-3β/3α			
1.	Kadsuranin (33)	Both	Reduce cognitive impairments. Increased liver glycogen. Block p-GSK-3β/p-tau axis.
2.	34	*In-vitro* only	Reduce Tau phosphorylation.
3.	35	*In-vivo* only	Reduce Tau phosphorylation.
4.	59	Both	Reduce cytokines, APP and p-tau. Enhance p-GSK-3β. Improve behavior.
5.	OCM-51 (36)	*In-vitro* only	Reduce phosphorylated Tau.
6.	PT-65 (62)	Both	Reduce phosphorylated Tau. Improve dementia.
Targeting HDAC6			
7.	38	*In-vivo* only	Improve memory problems.
8.	39	Both	Prevent phosphorylated tau & aggregation.
Targeting Dyrk1A			
9.	Dp-Findy (37)	*In-vitro* only	Reduce Dyrk1A.
Targeting JNK3			
10.	43 44	*In-vivo* only	Improve cognitive memory.
Targeting AChE and BuChE			
11.	45	*In-vivo* only	Improve learning and cognitive deficiencies.
12.	F681-0222 (47)		Reduce soluble Aβ42.
13.	48	Both	Block tau phosphorylation. Improve cognitive impairment.
14.	49		Reduce phosphorylated Tau. Reduce oxidative stress.
15.	50	*In-vitro* only	Inhibits inflammation.
16.	52	*In-vivo* only	Improve memory.
17.	55		Less agonism
Targeting PDK-1			
18.	PS-48 (57)	Both	Elevate Akt. Restore the inhibition of LTP.
Targeting Trk			
19.	ACD856 (58)	*In-vivo* only	Improve memory & cognitive function.
Targeting Aβ-LilrB2			
20.	ALI6 (65)	*In-vitro* only	Block Aβ binding to nerves. Restore p-cofilin.
21.	66		Restore p-cofilin. Tau hyperphosphorylation.
Targeting TTR-Aβ			
22.	Iododiflunisal (67)	*In-vitro* only	Decrease caspase-3
23.	Luteolin (68)		
24.	Sulindac (69)		
25.	Olsalazine (70)		
26.	Flufenamic (71)		
Targeting Keap1-Nrf2			
27.	NXPZ-2 (72)	*In-vivo* only	Improve memory deficits.
28.	POZL (73)		Reduce oxidative stress

GSK-3β/3α, Glycogen Synthase Kinase-3β/3α; HDAC6, Histone deacetylase 6; TTR-Aβ, Transthyretin-Aβ; Keap1, Kelch-like ECH-associated protein 1; Nrf2, Nuclear factor erythroid related factor 2; Aβ-LilrB2, Amyloid β linked Leukocyte Immunoglobulin-Like Receptor Subfamily B Member 2; LTP, Long term potential; Dyrk1A, Dual-specificity tyrosine-(Y)-phosphorylation-regulated kinase 1 A. Compound codes presented in this table suggest “names were not allotted to them because they are evaluated at the preclinical level only”. These drugs were validated using multi-omics approaches.

### 8.1 Selective/specific inhibition

Concerns regarding the toxicity and adverse effects of pan-inhibitors, along with a greater comprehension of the functions of disease-related proteins in normal bodily functioning, have led to significant advancements in the creation of selective inhibitors. By specifically targeting particular protein classes, subtypes, or structural regions, these innovative inhibitors may increase patient efficacy, safety, and tolerance (211, 212). By competing with ATP, two stereoisomers of Schisandra chinensis fruits, Kadsuranin [(+)-2] and Gomisin N [(−)-2], efficiently inhibit GSK-3β (213). Studies have shown that using these compounds can help with memory issues and significantly lower the levels of phosphorylated tau in the hippocampus regions of APP/PS1 double-transgenic mice. Interestingly, concentrating on the less conserved substrate binding sites rather than the ATP binding sites may increase drug selectivity, functional control, and safety. For instance, Compound 34 has demonstrated these benefits (214–216). Since GSK-3α is crucial for raising Aβ production and tau phosphorylation in AD animals, blocking it appears to be a successful therapy approach. The ATP-competitive GSK-3α inhibitor (compound 35) can cross the blood-brain barrier and dramatically lower tau phosphorylation at the pThr231 site in neonatal rat brains, which may delay the early beginning of AD pathogenesis. However, simultaneously inhibiting GSK-3α and GSK-3β may overactivate the wnt/β-catenin pathway, leading to increased cell proliferation and other adverse effects (215–217).

The goal of selective drugs is to achieve the best possible balance between safety and efficacy, making sure that they work as intended without endangering patients. OCM-51, a selective inhibitor of GSK3β, prevents overactivation of the β-catenin signaling pathway while lowering tau phosphorylation, a critical component in AD. Targeting the dynamic alterations in disease-related proteins is a further intriguing tactic (218). For example, by affecting mechanisms like tau hyperphosphorylation, APP and PS1 regulation, and tau premRNA splicing, overexpression of the protein DYRK1A can aid in the early onset of AD. By selectively targeting the folding intermediate state, the molecule dp-FINDY takes advantage of structural changes in the ATP-binding site of DYRK1A. This approach lessens unnecessary disruption to the protein’s normal functions while creating new opportunities for developing more targeted and accurate therapies (219–221).

HDAC inhibitors: By eliminating acetyl groups from protein lysine residues, HDACs control the expression of genes. Numerous biological processes, such as cell proliferation, differentiation, and development, are regulated by this system. Two catalytic domains and a zinc finger domain at the C-terminus make HDAC6 unique (222, 223). HDAC6 breaks down protein clumps, promotes mitochondrial transport, and improves cognitive memory via interacting with tau and α-tubulin. These roles are pertinent to AD (224–226). Three parts are commonly found in HDAC6 inhibitors: a zinc-binding group (ZBG), a cap group, and a hydrocarbon bond between them. A common basis for their specificity is the strong hydrophobic interactions between the cap group and a unique structural characteristic of HDAC6 called the “L1 loop pocket.” Notably, compound 38 demonstrated enhanced selectivity for HDAC6 due to the presence of a cap group produced from melatonin and ferulic acid. Even at lower dosages, it significantly enhanced antioxidant effects and improved spatial working memory and non-spatial long-term memory deficits in mice injected with Aβ25-35 (225, 227–229).

Compound 39 was developed to specifically target HDAC6 and decrease tau hyperphosphorylation and aggregation, two significant processes linked to AD, by interacting with a specific location on the protein. Through the modulation of ubiquitination pathways, it also shown neuroprotective benefits and improved learning and memory in animal trials, making it a promising treatment option for AD. When developing selective inhibitors, researchers often start with a lead molecule that provides a basic structure and binding affinity. They then alter the chemical to improve its ability to bind to its target as well as its solubility, stability, and blood-brain barrier crossing capabilities (230). For instance, a combination of computer-based screening and pharmacophore modeling was used to identify compounds 40 and 41 from an existing cancer chemical library. In order to develop new inhibitors that target casein kinase 1δ (CK1δ), another protein implicated in disease processes, their shared structural framework should be helpful (231).

Activation of JNK3 is essential for tau tangle development, amyloid buildup, and neuronal injury in AD (232). This target has been the subject of extensive research by Hah et al., who have continuously improved and produced numerous generations of medications derived from pan-JNK inhibitor 42, which was discovered through specialized kinase-focused screening. These new compounds have shown significant gains in pharmacokinetics, potency, and selectivity while preserving important interactions with JNK3 (233–235).

Using two distinct mouse models of AD—the 3xTg dementia model and the APPswe/PS1dE9 double transgenic mice—currently researched chemicals 43 and 44 showed remarkable behavioral study outcomes. These results explore the possibility of selective inhibitors, which reduce the likelihood of adverse effects and are more useful in helping researchers identify the precise targets in AD and its subtypes. By focusing on selectivity, the numerous adverse effects that result from mixing medications with undesirable proteins can be avoided. By focusing on differences in amino acids, the presence of water molecules, and the structural and dynamic properties of protein binding sites, researchers can develop treatments that are more precise and effective while reducing the likelihood of off-target consequences (235).

When developing therapies for AD, achieving high selectivity for inhibitors is a major challenge, especially when dealing with largely structurally similar binding sites. Utilizing a variety of computational techniques, optimizing targets (by identifying substitute or alternative locations), and exploring novel pockets on the target enzyme may all result in the creation of new chances. The complexity and diversity of AD pathways make it difficult to focus on specific targets, and their influence on the progression of the disease may remain limited. By depending on precise distribution and functionality during the pathological stage of the disease, pharmacological approaches aim to improve efficacy and reduce side effects in addition to targeting specific enzymes. Numerous examples exist, such as the intriguing potential of PROTAC technology for the treatment of AD. It works by triggering the targeted degradation of particular proteins by activating E3 ligases, which may be expressed selectively in particular tissues. Covalent medications also have a lot of potential for selective targeting. These medications could introduce new methods for blocking kinases like CK1, which are currently solely inhibited by non-covalent ATP-competitive inhibitors. Drug development advancements are anticipated to increase therapy efficacy and safety while expanding the range of selectivity available for AD treatments (236).

### 8.2 Dual-target inhibitors

Single-target drugs usually don’t work as a successful treatment for AD, which has multiple contributory factors at first. As a result, scientists are currently developing inhibitors that target one or more targets. These therapies target many pathways, either enhancing their effects or collaborating to get superior outcomes (237). The goals are to decrease side effects, boost efficacy, prolong the duration of benefits, and minimize dosages. These multi-target medicines with combination therapy also simplify treatment and lessen the likelihood of drug interactions, making them safer, more effective, and easier for patients to use. At the molecular level, there is mounting evidence linking cholinergic system disruption to abnormal Aβ and tau accumulation, two other significant characteristics of AD (238, 239).

Cholinesterase-inhibiting medications have become an essential part in treating AD. Both AChE and butyrylcholinesterase (BuChE) are targeted by these inhibitors, which act as disease-modifying medications by reducing the formation of Aβ plaques and enhancing cognitive abilities in AD patients by raising acetylcholine levels (240, 241). The compound 45, for instance, has shown remarkable results in inhibiting AChE and BuChE in aged AD animals. The substance greatly enhanced learning and memory, enhances synaptic communication in the hippocampus, and possesses anti-inflammatory and antioxidant qualities. Moreover, a possible treatment for AD is to boost the activity of MAO-B by blocking both AChE and MAO-B. This multimodal approach has synergistic effects for treating AD by reducing oxidative stress, repairing the cholinergic system, and lowering the production of Aβ plaque (242–244). Ladostigil, a new AChE and MAO-B inhibitor created by pharmacophore fusion, just concluded a Phase II clinical trial (NCT01429623). This study aimed to assess the safety and effectiveness of low-dose medication in people with MCI. The results indicated that the medication was safe, well-tolerated, and had encouraging potential for delaying the progression of AD (245, 246).

The chemical F681–0222 was designed to prevent the interaction between BACE1 and AChE. In the brain tissue of the APPswe/PS1dE9 transgenic mice, it reduced the levels of soluble Aβ42 (247). Concurrently, it has shown promise in regulating tau protein signaling and boosting cholinergic function by focusing on the GSK-3β and AChE pathways. In ICR mice given scopolamine, compounds 48 and 49, which were made using the pharmacophore linking approach, demonstrated promise in lowering tau hyperphosphorylation and alleviating cognitive deficits (248). Additionally, phosphodiesterases (PDEs) have demonstrated promise in improving synaptic function and reducing cognitive deficits when used to treat AD (249, 250). In the hippocampus of AD mice treated with Aβ25-35, compound 50, an inhibitor of both AChE and PDE4D, demonstrated higher anti-inflammatory qualities and superior neuroprotective benefits against cell death than a combination treatment of Donepezil (compound 4) and Rolipram (compound 51). This demonstrates its potential as a very effective AD treatment option (251).

Sometimes medications that target a single aspect of a disease, like Alzheimer’s, don’t function effectively for illnesses with complex etiologies. In addition to perhaps failing to appropriately treat the intricate web of factors underlying the illness, they can occasionally cause catastrophic side effects. The development of dual-target and multi-target inhibitors is the focus of increased research to address this. The connections between different illness systems are taken into consideration when creating these drugs, which enhances patient convenience and treatment effectiveness. Several interconnected AD pathways are influenced by multi-target drugs. Although their combined effect may not be as effective on any one target as single-target drugs, it is still stronger than the sum of their separate effects. In many cases, this combination leads to better overall results and fewer negative effects (252).

These medications can be made in two main ways: either by joining pharmacophores (a drug’s active ingredients) or by combining them. Although the design process is made simpler by these tactics, the range of drug structures and targets that can be addressed may be constrained. Researchers usually go to computer-based screening or natural substances to remedy this. But developing such drugs entails more than just focusing on multiple pathways. Assessing the drug’s pharmacokinetics, possible toxicity, and physical and chemical properties is also essential. Developing safe and effective medications requires striking a balance between all of these factors.

### 8.3 Allosteric inhibition

Allosteric modulators work by binding to parts of a receptor that are not the primary (orthosteric) binding site. This allows them to modify receptor activity, which can either directly change the function of the receptor (raising, decreasing, or neutralizing its effects) or indirectly change the binding efficiency of other molecules. This tactic has reignited interest in developing drugs that inhibit γ-secretase in AD. These modulators help realign γ-secretase activity to Favor the creation of shorter, less dangerous Aβ proteins, potentially reducing unwanted effects on Notch and other substrates (253–255). Some γ-secretase modulators (GSMs) have also shown promising safety results in preclinical and clinical trials (256, 257). Particularly for closely related receptor subtypes like nAChR and mAChR, allosteric sites are promising targets for drug development because they are less conserved and more varied than orthosteric sites. The α7 nAChR subtype in particular is being considered as a potential target for AD treatment due to its high frequency in brain regions essential for cognitive function and its interactions with Aβ (258, 259). Compound 52 and other selective positive allosteric modulators (PAMs) that target the α7 nicotinic acetylcholine receptor (α7 nAChR) subtype have shown promise in lowering working and episodic memory loss in animal models of amnesia. Unlike traditional orthosteric agonists, compound 52 does not desensitize receptors, even after repeated treatment. Its effectiveness and safety in those with mild to moderate AD are now being assessed through clinical trials (260).

To achieve subtype selectivity, M1 muscarinic acetylcholine receptor (M1-mAChR) PAMs, such as BQCA (compound 53) and PF06764427 (compound 54), employ allosteric mechanisms. However, one of the adverse consequences that could possibly arise from their powerful agonistic activity is diarrhoea. Further *in vitro* and *in vivo* studies are necessary to investigate the pharmacokinetics and allosteric modulation properties of compounds 55 and 56, which are optimized derivatives of these drugs. Additionally, achieving signaling bias by allosteric modulation is a crucial research topic in the development of safer M1-mAChR drugs. As it may improve their therapeutic profile and reduce side effects, this approach is crucial for developing strong M1-mAChR allosteric ligands (255, 260).

Allosteric drugs are being thoroughly researched for AD, in addition to concentrating on the cholinergic system (261). For instance, PS48 (57) binds to an allosteric location on PDK-1 to restore insulin responsiveness in Akt. By reducing the negative consequences of Aβ without overstimulating insulin signaling, this approach presents a promising means of treating or preventing AD. In a phase I clinical trial, the new allosteric modulator ACD856 (58), which targets Trk receptors, showed favorable pharmacokinetic properties, acceptable safety, and tolerability (NCT05077501). The ability of this drug to enhance neurotrophic factor signaling may be advantageous for AD patients. Allosteric modulation approaches have also been extensively studied for other proteins implicated in AD, including as RIPK1, NMDA receptors, AMPA receptors, and GSK-3β, as mentioned in several studies (262, 263).

These techniques provide up new possibilities for the creation of targeted and effective treatments. Allosteric modulation comes out as a very attractive method in the therapy development process for AD due to its unique properties. Examples include producing saturated allosteric effects, fine-tuning target protein activity, targeting low conservation binding sites, and offering subtype or pathway selectivity. Despite its potential, the process of discovering and developing allosteric drugs has a unique set of challenges. Advances in techniques like molecular docking, dynamics simulations, X-ray crystallography, and cryo-electron microscopy have significantly improved our ability to identify allosteric sites and deepen our understanding of allosteric mechanisms. However, because of the complexity of this technique, a significant amount of *in vitro* and *in vivo* research is required to fully evaluate the functional impact of these drugs and determine the factors controlling their behavior. Despite the great promise for allosteric drugs to alleviate the cognitive deficits associated with AD, more comprehensive experimental evidence is required to ascertain their efficacy and safety in order to guide future research and development (256).

### 8.4 Covalent inhibitors

Covalent inhibitors may form precise and stable bonds with target proteins, they offer significant advantages such as enhanced potency, selectivity, and longer-lasting effects. They are a convenient therapeutic choice for patients because of this process. Given their effectiveness in treating cancer and other diseases, covalent inhibitors hold great potential for the creation of medications to treat AD. Acrylamide warheads are commonly used by these inhibitors to target cysteine residues in cancer treatment (264, 265). Building on this strategy, chemical 59 efficiently binds covalently to cysteine in GSK-3β thanks to its acrylamide warhead design. It has been demonstrated to dramatically lower phosphorylated tau and APP levels in the hippocampal regions of AD mice, improving memory and spatial learning (193). Meanwhile, a new anti-Aβ strategy is provided by the anti-cancer drug KP1019 (compound 60), which is based on Ru (III). Unlike conventional methods that focus on blocking Aβ synthesis or aggregation, KP1019 counteracts Aβ toxicity in neuronal cell models by encouraging the formation of soluble, high-molecular-weight Aβ aggregates. This unique process offers a fresh method for creating therapeutics for AD. Further, metal-based covalent inhibitors are showing promise as AD treatments (266). Researchers are continuously refining these inhibitors by strengthening their reactive components (electrophilic warheads) and their capacity to target specific residues. For instance, NMR screening was used to identify the 6H8 (61) fragment from the Maybridge collection. It might act as a covalent warhead directed at APP, an essential substrate of γ-secretase. By interfering with APP processing, this tactic may help reduce Aβ formation and maybe circumvent some of the side effects of γ-secretase inhibitors (266, 267). Additionally, covalent inhibitors have expanded the range of potential treatments by making previously “undruggable” targets—such as Aβ, tau, and APPTM more accessible for drug development. By lowering the necessary dosage and frequency of administration, their long-term benefits may enhance patient adherence to therapy. However, concerns about potential toxicity remain. To improve the selectivity of covalent inhibitors, researchers are investigating methods to optimize their design. This means adjusting dosage levels, changing non-covalent scaffolds, and fine-tuning the reactivity and reversibility of the electrophilic warhead. These strategies are being intensively studied by the scientific community (268).

### 8.5 PROTACs

Ubiquitin-proteasome system (UPS) plays a major role in cellular protein degradation. Failure in this system, however, is primarily responsible for the accumulation of harmful proteins in AD (269). Protein clearance is made more accurate and efficient by PROTACs, which use the UPS system to specifically target and eliminate specific proteins. Several analyses have highlighted the potential applications of PROTACs in AD (270). These drugs specifically target tau, the transthyretin (TTR)-Aβ connection, HDACs, BET proteins, and the phosphokinase GSK-3β, among other proteins associated with disease. Among the notable advantages they offer are low dosage requirements, outstanding potency, and remarkable target selectivity (269, 270).

As technology develops, PROTACs keep evolving. The click chemistry-produced GSK-3 degrader PT-65 (compound 62) has shown a longer-lasting effect on phosphorylated tau (p-tau) than its GSK-3 warhead, a traditional GSK-3 inhibitor. Its extended effect, which may reduce the frequency of required doses, could further increase its therapeutic attractiveness (271). PROTACs cleverly leverage their flexible structure to develop targeted strategies for removing phosphate groups from proteins, such as tau2-8 (64) (272) and phosTAC7 (63) (273). Briefly stated, PROTACs are a new and innovative approach to the creation of drugs for AD. They work by targeting defective enzymes, misfolded proteins, and disease-causing protein-protein interactions with the body’s natural protein-clearing machinery, the ubiquitin-proteasome system, or UPS. However, there are still several challenges that PROTACs must face. Although E3 ligases such as VHL and CRBN are widely utilized in PROTAC design, their limited repertoire can constrain ligand flexibility and target specificity. In addition, the relatively large molecular size of PROTACs often impairs BBB permeability, posing a significant challenge for the treatment of central nervous system disorders such as AD. Moreover, while PROTACs can selectively degrade existing disease-associated proteins, they cannot reverse irreversible downstream pathological damage that has already occurred. This limitation is particularly relevant in the context of neurodegenerative conditions and genetic forms such as familial Alzheimer’s disease (FAD), where early molecular and cellular alterations may already have progressed beyond the point of therapeutic reversal. Despite these challenges, PROTACs have considerable promise as a new weapon in the fight against AD (269).

### 8.6 Targeting the PPI network

Although the maintenance of biological processes depends on PPIs, aberrant PPIs have been linked to the development of many diseases. For instance, tau and Aβ proteins in AD misfold and aggregate due to complex PPI networks and complex biochemical pathways; by disrupting these detrimental relationships, it may be possible to halt key signaling pathways and slow the disease’s progression (274, 275).

Even though peptide and protein-based drugs are usually better suited for concentrating on the large, flat interfaces of PPIs, small-molecule inhibitors offer unique advantages in treating particular AD-related PPIs. For example, Aβ interacts with the leukocyte immunoglobulin-like receptor B2 (LilrB2) to negatively impact memory and synaptic function (276, 277). Compounds ALI6 (65) and 66 have been shown to effectively break this link, reestablishing cofilin signaling downstream of LilrB2 and promoting neurite growth. By protecting neuronal cells from the damaging effects of Aβ, these compounds offer a potential therapy option for AD. Transthyretin (TTR) and Aβ have a beneficial relationship because it lessens Aβ toxicity and aggregation. This interaction is facilitated by a number of small-molecule chaperones, such as luteolin, iododiflunisal (IDIF), and three modern medications: Flufenamic acid, Olsalazine, and sulindac. It has been shown that these substances significantly lower SH-SY5Y cell death by reducing caspase-3 activation. They are also intriguing prospects for confirming TTR as a therapeutic target and examining its potential in AD clinical trials due to their high BBB crossing capacity (278, 279). Another fascinating PPI target is the Keap1-Nrf2 pathway, which is crucial for regulating the body’s antioxidant response (280). NXPZ-2, an oral covalent inhibitor of this pathway, has been shown to improve cognitive function in mice with Aβ-induced impairments by upregulating Nrf2 expression and its downstream antioxidant defences. However, concerns were initially raised by problems including poor solubility and little validation in transgenic AD models. These issues were effectively resolved by developing a superior counterpart with greater potential for medical use (281). The structural features of the Keap1-Nrf2 ETGE complex are replicated by compound 74, another prospective inhibitor of the Keap1-Nrf2 PPI. A new inhibitory mechanism has been discovered by this creative approach, opening up a new avenue for the creation of unique Keap1-Nrf2 PPI inhibitors (282).

In summary, focusing on key pathogenic linkages and either activating or inhibiting them is a potential approach to treating AD. PPI modulators work precisely and reversibly by carefully and gently focusing on disease-related pathways. Therefore, they are a safer option since they can preserve proteins’ normal functions while reducing the likelihood of severe side effects. Furthermore, recent advancements in computational tools and modeling approaches have made it easier to identify extremely effective and precise PPI-targeting drugs. Because PPIs are dynamic and transient, these methods let researchers find underutilized or optimal protein interaction locations. This opens up exciting new possibilities for the creation of cutting-edge and successful PPI-based treatments (283, 284).

## 9 AI-enabled precision medicine: multi-omics integration and digital biomarkers

The marked biological and clinical heterogeneity of AD presents a major challenge to the development of effective disease-modifying therapies. Patients with clinically similar symptoms often exhibit distinct molecular signatures involving amyloid pathology, tau aggregation, neuroinflammation, mitochondrial dysfunction, vascular injury, and metabolic abnormalities. Consequently, precision medicine approaches that integrate multi-dimensional biological information are increasingly being recognized as essential for improving diagnosis, patient stratification, and therapeutic decision-making. Recent advances in multi-omics technologies including genomics, epigenomics, transcriptomics, single-cell transcriptomics, proteomics, metabolomics, lipidomics, microbiomics, and spatial transcriptomics, provide complementary insights into the complex molecular architecture of AD. Integrating these datasets enables reconstruction of disease-associated molecular networks and facilitates identification of patient-specific pathogenic pathways that cannot be detected using single-omics analyses alone (285–289).

Artificial intelligence (AI), particularly machine learning, deep learning, graph neural networks, and knowledge graph-based approaches, has emerged as an indispensable tool for analysing these high-dimensional datasets. AI algorithms can integrate heterogeneous molecular, imaging, and clinical information to identify latent disease subtypes, predict disease progression, discover novel biomarkers, and prioritize therapeutic targets. Network-based AI approaches have further accelerated drug repurposing by identifying previously unrecognized drug–target interactions and signaling pathways involved in neuroinflammation, microglial activation, and tau pathology. In addition, generative AI and structure-based deep learning models are increasingly being applied to virtual screening, *de novo* drug design, prediction of protein–ligand interactions, and optimization of lead compounds, thereby reducing both the cost and duration of traditional drug discovery pipelines (290–292).

An important application of multi-omics integration is biomarker-guided precision medicine. Rather than relying on individual biomarkers, AI models combine plasma biomarkers (Aβ42/Aβ40, phosphorylated tau species, neurofilament light chain, and GFAP), cerebrospinal fluid biomarkers, neuroimaging parameters, genetic risk variants such as APOE genotype, and molecular omics profiles to classify patients into biologically meaningful subgroups. Such stratification enables identification of patients most likely to benefit from anti-amyloid therapies, anti-inflammatory interventions, or emerging disease-modifying treatments while reducing unnecessary exposure to ineffective therapies. Multi-modal predictive models also improve prognosis by estimating rates of cognitive decline, conversion from mild cognitive impairment to dementia, and therapeutic response (293, 294).

Beyond conventional biomarkers, digital biomarkers are emerging as valuable tools for continuous and non-invasive assessment of disease progression. Digital biomarkers are objective physiological or behavioral measurements collected using smartphones, wearable devices, speech analysis, eye-tracking systems, smart-home sensors, gait monitoring, sleep assessment, and cognitive testing platforms. AI algorithms analyse these longitudinal datasets to detect subtle alterations in cognition, language, mobility, sleep quality, and daily activities that frequently precede clinically detectable cognitive impairment. Continuous digital monitoring therefore complements traditional neuroimaging and fluid biomarkers by enabling real-time disease surveillance, remote patient monitoring, and early therapeutic intervention. Integration of digital biomarkers with molecular biomarkers is expected to improve diagnostic sensitivity and facilitate personalized disease management (295–298).

Another promising development is the emergence of AI-enabled digital twin technology. Digital twins are computational models that integrate longitudinal clinical information, neuroimaging, multi-omics datasets, and digital biomarkers to generate individualized virtual representations of patients. These models can simulate disease progression, predict treatment responses, identify optimal therapeutic combinations, and support adaptive clinical trial design. Although still in early stages of development, digital twins represent a promising framework for precision drug discovery and personalized treatment optimization in AD (299–301).

Despite these advances, several challenges remain before AI-assisted precision medicine can be routinely implemented in clinical practice. Multi-omics datasets are often heterogeneous, generated using different experimental platforms, and limited by relatively small sample sizes and incomplete longitudinal follow-up. AI models frequently function as “black boxes,” limiting biological interpretability and clinician confidence. Additional barriers include insufficient external validation across ethnically diverse populations, lack of standardized data harmonization pipelines, regulatory considerations, algorithmic bias, patient privacy concerns, and integration into existing healthcare workflows. Addressing these challenges through large multicentre cohorts, standardized data-sharing frameworks, explainable AI, and prospective clinical validation will be essential for translating AI-enabled precision medicine into routine clinical care for AD (302–304).

## 10 Strengths, limitations, and unresolved challenges in AD research

Substantial progress has been made in understanding the molecular mechanisms underlying AD, particularly the interplay among Aβ, tau pathology, neuroinflammation, mitochondrial dysfunction, vascular impairment, and metabolic dysregulation. The development of highly sensitive fluid biomarkers, advanced neuroimaging techniques, and disease-modifying therapies has transformed the diagnostic and therapeutic landscape, enabling earlier detection of pathological changes and more accurate biological classification of patients. Furthermore, recent advances in single-cell and spatial transcriptomics, multi-omics integration, and artificial intelligence have expanded our understanding of disease heterogeneity and facilitated the identification of novel therapeutic targets (305–307).

Despite these advances, several major controversies remain unresolved. One of the most debated issues is the clinical benefit of anti-amyloid therapies (53). Although monoclonal antibodies targeting aggregated Aβ, including lecanemab and donanemab, consistently reduce cerebral amyloid burden and modestly slow cognitive decline in patients with early AD, the magnitude of clinical benefit remains relatively small compared with the substantial reduction in amyloid pathology. In addition, treatment is associated with ARIA, including cerebral edema (ARIA-E) and micro-haemorrhages (ARIA-H), which require regular MRI monitoring and careful patient selection. These findings suggest that amyloid removal alone may be insufficient to halt disease progression once extensive tau pathology, neuroinflammation, synaptic dysfunction, and neuronal loss have become established (112, 156, 158).

Another important challenge is the biological heterogeneity of AD. Increasing evidence indicates that AD represents a spectrum of molecular subtypes rather than a single disease entity. Patients differ substantially in genetic risk factors, inflammatory responses, vascular pathology, metabolic dysfunction, and rates of disease progression. Consequently, therapies demonstrating efficacy in one subgroup may show limited benefit in others. The lack of robust patient stratification has likely contributed to the high failure rate of clinical trials over the past two decades (308, 309).

Although biomarkers have markedly improved diagnostic accuracy, important limitations remain. Amyloid PET may detect Aβ deposition in cognitively normal older adults, reducing its specificity for symptomatic AD, whereas tau PET, although more closely associated with cognitive decline, remains limited by cost, availability, and off-target tracer binding. CSF biomarkers provide excellent diagnostic performance but require lumbar puncture and remain susceptible to pre-analytical variability (23, 113, 120). Plasma biomarkers, particularly phosphorylated tau isoforms (p-tau181, p-tau217, and p-tau231), have emerged as promising, minimally invasive tools for screening and disease monitoring. However, differences among analytical platforms, assay standardization, biological variability, and the influence of comorbid conditions such as chronic kidney disease, cerebrovascular disease, and systemic inflammation continue to limit universal clinical implementation. Therefore, multimodal diagnostic approaches integrating imaging, fluid biomarkers, genetics, and clinical assessment remain essential (23).

The limited translation of promising preclinical findings into successful clinical therapies remains another major obstacle. Numerous therapeutic strategies targeting neuroinflammation, oxidative stress, mitochondrial dysfunction, and protein aggregation have demonstrated encouraging efficacy in cellular and animal models but have failed to produce meaningful clinical benefit in humans. These translational failures likely reflect limitations of conventional animal models, which incompletely reproduce the complex pathology, aging processes, and genetic diversity observed in human AD (310, 311). Moreover, most experimental models do not fully recapitulate chronic neuroinflammation, mixed proteinopathies, cerebrovascular pathology, or age-related immune dysfunction that characterize sporadic AD (312).

The precise temporal relationship between amyloid deposition, tau propagation, neuroinflammation, vascular dysfunction, and neuronal degeneration remains incompletely understood. Whether neuroinflammation primarily initiates disease, accelerates ongoing pathology, or exerts stage-dependent protective and detrimental effects continues to be actively investigated. Similarly, the mechanisms governing the transition of microglia and astrocytes between homeostatic and disease-associated phenotypes require further clarification. Improved understanding of these processes will be critical for identifying optimal therapeutic windows and designing interventions that selectively modulate beneficial rather than detrimental immune responses (313–315).

Future therapeutic development is therefore likely to shift from single-target interventions toward personalized, multimodal treatment strategies (316, 317). Combining anti-amyloid or anti-tau therapies with agents targeting neuroinflammation, mitochondrial dysfunction, metabolic abnormalities, or vascular impairment may provide greater clinical benefit than monotherapy (318). Integration of multi-omics datasets, blood-based biomarkers, digital biomarkers, and artificial intelligence-driven patient stratification is expected to facilitate precision medicine approaches that identify patients most likely to benefit from specific therapies (319). Continued international collaboration, standardized biomarker validation, and adaptive clinical trial designs will be essential for translating mechanistic discoveries into effective disease-modifying treatments for Alzheimer’s disease.

## 11 Conclusions and future perspectives

AD remains a complex and multifactorial neurodegenerative disorder, primarily characterized by memory loss and cognitive decline. While hallmark pathological features such as NFTs and Aβ plaques have been widely studied, their precise roles in disease onset and progression remain only partially understood. Emerging evidence underscores the significance of the immune system and neuroinflammatory responses, particularly involving microglial activation and innate immune signaling, in the pathophysiology of AD. Intriguingly, certain pathogens such as *Porphyromonas gingivalis* and herpes simplex virus 1 (HSV1) have been implicated in modulating these responses, suggesting a potential infectious component to disease development. These findings warrant further investigation into the interplay between infection, neuroinflammation, and amyloid/tau pathology (**Figure 7**).

**Figure 7:**
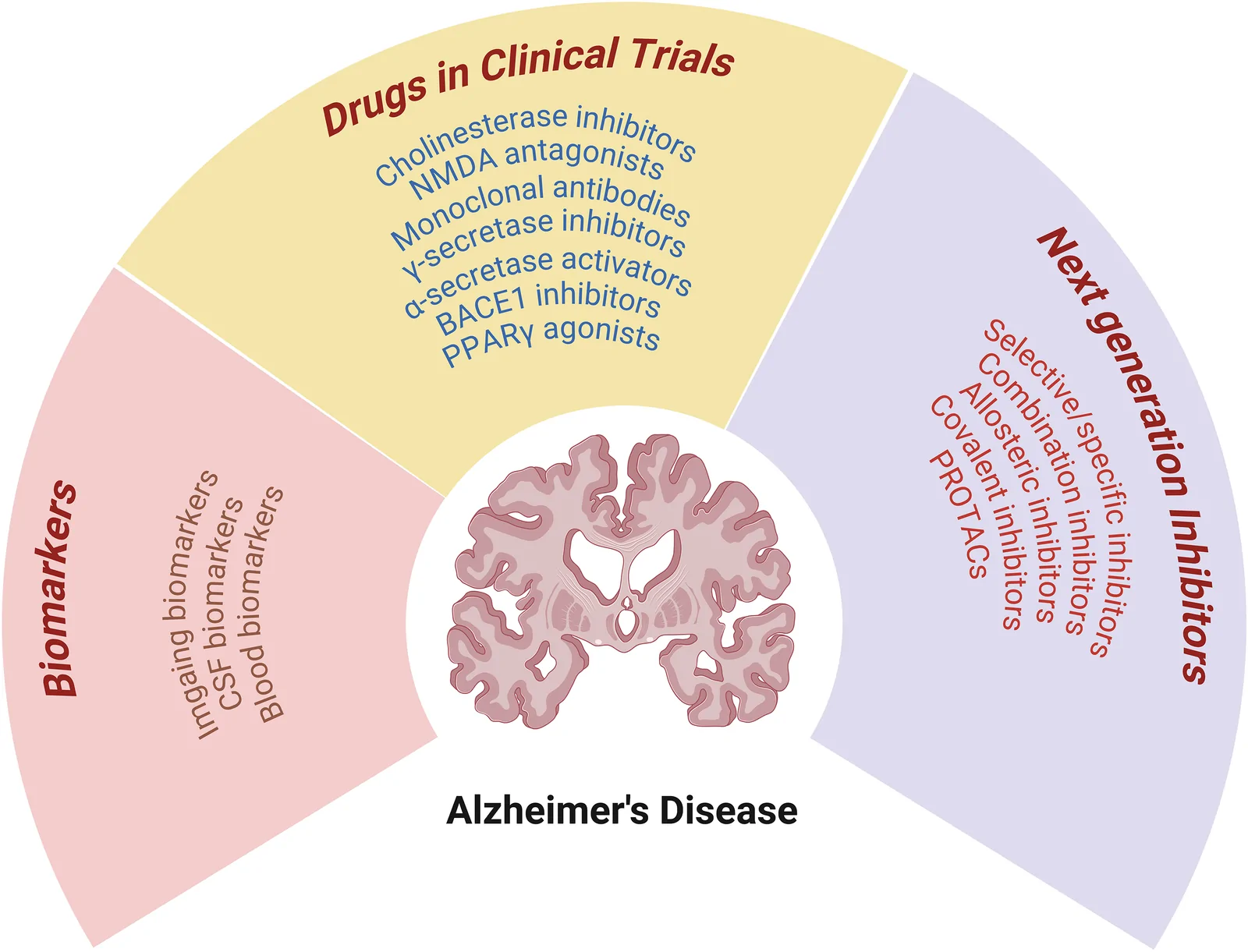
Alzheimer’s disease biomarker identification, drugs in clinical trials and next-generation inhibitors.

A major emphasis in AD research continues to be on early detection, prevention, and the development of effective therapeutic interventions. The identification and validation of reliable biomarkers—found in blood, CSF, saliva, urine, and even the retina—represent significant progress in enabling earlier diagnosis, monitoring disease progression, and predicting therapeutic responses. These less invasive and more accessible diagnostic tools hold great promise for improving clinical outcomes and streamlining patient stratification in clinical trials. Therapeutic strategies remain a challenge, with pharmacologic treatments often limited by issues such as low BBB permeability, dose-limiting side effects, and individual variability in drug metabolism and responsiveness. A deeper understanding of disease mechanisms is essential to guiding therapeutic decisions.

Looking forward, the integration of advanced technologies such as AI is expected to revolutionize AD research. AI-driven platforms can accelerate biomarker discovery, optimize drug development pipelines, predict disease trajectories, and personalize treatment strategies based on complex datasets. Strategic drug development approaches that incorporate rational target selection, detailed mechanism-of-action analyses, and precision pharmacology will be instrumental in overcoming existing therapeutic barriers. By combining these advancements with a systems-level understanding of AD pathology, researchers and clinicians can move closer to delivering safe, effective, and individualized treatments for patients. Continued interdisciplinary research, along with robust clinical validation of emerging biomarkers and therapeutic targets, will be critical to transforming the current landscape of AD management.
